# Wild bees of Grand Staircase-Escalante National Monument: richness, abundance, and spatio-temporal beta-diversity

**DOI:** 10.7717/peerj.5867

**Published:** 2018-11-07

**Authors:** Olivia Messinger Carril, Terry Griswold, James Haefner, Joseph S. Wilson

**Affiliations:** 1 Santa Fe, NM, United States of America; 2 USDA-ARS Pollinating Insects Research Unit, Logan, UT, United States of America; 3 Biology Department, Emeritus Professor, Utah State University, Logan, UT, United States of America; 4 Department of Biology, Utah State University - Tooele, Tooele, UT, United States of America

**Keywords:** Bee declines, Pollinators, Conservation, National monuments, Native bees, Pollination ecology

## Abstract

Interest in bees has grown dramatically in recent years in light of several studies that have reported widespread declines in bees and other pollinators. Investigating declines in wild bees can be difficult, however, due to the lack of faunal surveys that provide baseline data of bee richness and diversity. Protected lands such as national monuments and national parks can provide unique opportunities to learn about and monitor bee populations dynamics in a natural setting because the opportunity for large-scale changes to the landscape are reduced compared to unprotected lands. Here we report on a 4-year study of bees in Grand Staircase-Escalante National Monument (GSENM), found in southern Utah, USA. Using opportunistic collecting and a series of standardized plots, we collected bees throughout the six-month flowering season for four consecutive years. In total, 660 bee species are now known from the area, across 55 genera, and including 49 new species. Two genera not previously known to occur in the state of Utah were discovered, as well as 16 new species records for the state. Bees include ground-nesters, cavity- and twig-nesters, cleptoparasites, narrow specialists, generalists, solitary, and social species. The bee fauna reached peak diversity each spring, but also experienced a second peak in diversity in late summer, following monsoonal rains. The majority of GSENM’s bees are highly localized, occurring in only a few locations throughout the monument, and often in low abundance, but consistently across the four years. Only a few species are widespread and super-abundant. Certain flowering plants appear to be inordinately attractive to the bees in GSENM, including several invasive species. GSENM protects one of the richest bee faunas in the west; the large elevational gradient, incredible number of flowering plants, and the mosaic of habitats are all likely contributors to this rich assemblage of bees.

## Introduction

Several recent analyses have concluded that certain species of wild bees may be in decline (e.g.,  Bartomeus et al., 2013; Goulson et al., 2015; Potts et al., 2010). Whether these declines are widespread across the more than 20,000 species found in the world is unknown (Ghazoul, 2015). Such a decline would pose a significant threat to both natural ecosystems and agricultural communities, as bees are the primary pollinators of most of Earth’s flowering plants (Ollerton, Winfree & Tarrant, 2011). Documenting the extent of declines both across populations of disparate bee taxa, and across widely differing ecosystems, however, has proven difficult for many reasons.

First, native bees vary naturally in population size from year to year (Williams, Minckley & Silveira, 2001; Wilson, Messinger & Griswold, 2009), making upward or downward trends difficult to establish without multiple consecutive years of data (Lebuhn et al., 2013). Second, bee communities are often comprised of a few common species and many rare species (e.g.,  Russo et al., 2011; Winfree et al., 2015), requiring that an inordinately large number of specimens be collected in order to document the majority that are present (Russo et al., 2015). Third, there are relatively few historic records documenting bee ranges (Gibbs et al., 2017). With so little data about the historic size of bee populations, it is difficult to demonstrate whether current numbers indicate an improvement or a deterioration in bee health. Few standardized bee collections were made prior to the last two decades (Bartomeus et al., 2013; Biesmeijer et al., 2006), so there are few baseline data points against which to measure present-day bee population sizes. Moreover, those few standardized collections that are older than twenty years are often in areas where the landscape has been significantly altered, so that collection events today may be sampling a much different habitat, from a bee’s perspective (Biesmeijer et al., 2006).

Protected lands, such as monuments and parks, provide an exceptional opportunity for monitoring bee populations and thoroughly documenting the presence and abundance of a bee fauna for a large area. As there is no mandate for multiple use, national monuments and national parks seldom experience large-scale, unnatural changes across a landscape (Acrygg et al., 2013; Stein, Scott & Benton, 2008), and are less affected by some of the potential threats to native bees, including pesticides and fertilizers (Roulston & Goodell, 2011; Woodcock et al., 2017), conversion to agricultural or otherwise developed lands (Forrest et al., 2015; Palma, 2015), competition with honey bee hives (Cane & Tepedino, 2017), and habitat fragmentation (Howell, Alarcon & Minckley, 2017; Steffan-Dewenter, 2003). They provide a valuable natural laboratory in which to conduct standardized collections, that allows for the resampling of an area over long periods to observe trends in bee populations. Finally, they provide a spotlight for the organisms within. Through outreach and education, monuments and parks enlighten millions of visitors each year about the creatures they protect, and the value of those creatures for ecosystem function (Fancy, Gross & Carter, 2009).

We conducted a large-scale, multi-year inventory of the bee fauna of one such area, Grand Staircase-Escalante National Monument (GSENM), which is a large area of protected land that, until recently, incorporated nearly 1.9 million acres of the Colorado Plateau in south-central Utah. While the monument is dominated by cold-desert plant communities, plants associated with warmer ecoregions occur at its southern and western extents. The region includes a diverse flora, with many endemic species (Fertig, 2005). Elevations in the monument range from 1,356 to 2,316 m, and encompass stands of aspen (*Populus tremuloides*), ponderosa (*Pinus ponderosa*), pinyon (*Pinus* spp.), juniper (*Juniperus* spp.), blackbrush (*Coleogyne ramosissima*), sagebrush (*Artemisia* spp.), grasslands, mixed desert scrub, meadows, and riparian zones. Temperatures range annually from −11 °C to 38 °C. Most precipitation occurs in the form of summer rainfall (average: 13.5 cm across the four years of our study, 2000–2003) with monsoonal weather events, but snow is also common throughout the monument from November through March (average across the years of the study: 11.7 cm) (Utah Climate Center, 2001–2005: https://climate.usu.edu/).

Our study of GSENM used standardized bi-monthly sampling in one-hectare plots, supplemented by opportunistic collections, in order to determine the richness and diversity of bees in this protected area, assess short and long-term population fluctuations in bee species, as well as associate bees with habitat types. Prior to our study, knowledge of the fauna was limited to only 20 collector-days of sampling for the monument, and all were along major roads (Griswold, Parker & Tepedino, 1998). Our study provides an example of the usefulness of national monuments and parks for scientific studies of insects whose populations are difficult to quantify. Eventually, our research will result in additional publications on the spatial and temporal drivers of bee diversity on this landscape.

For this particular paper, our focus is on documenting, summarizing, and broadly characterizing the diverse and unique bee fauna protected by GSENM. Specifically, we describe (1) the bee diversity, including new range boundaries and a summary of known life history traits, (2) spatial patterns, (3) seasonal patterns, and (4) floral relationships of the bee assemblage. Finally, we compare the diversity and composition of GSENM’s fauna with other known bee assemblages, and consider this community in the context of western bee distributions.

## Materials and Methods

### Field methods

**Figure 1:**
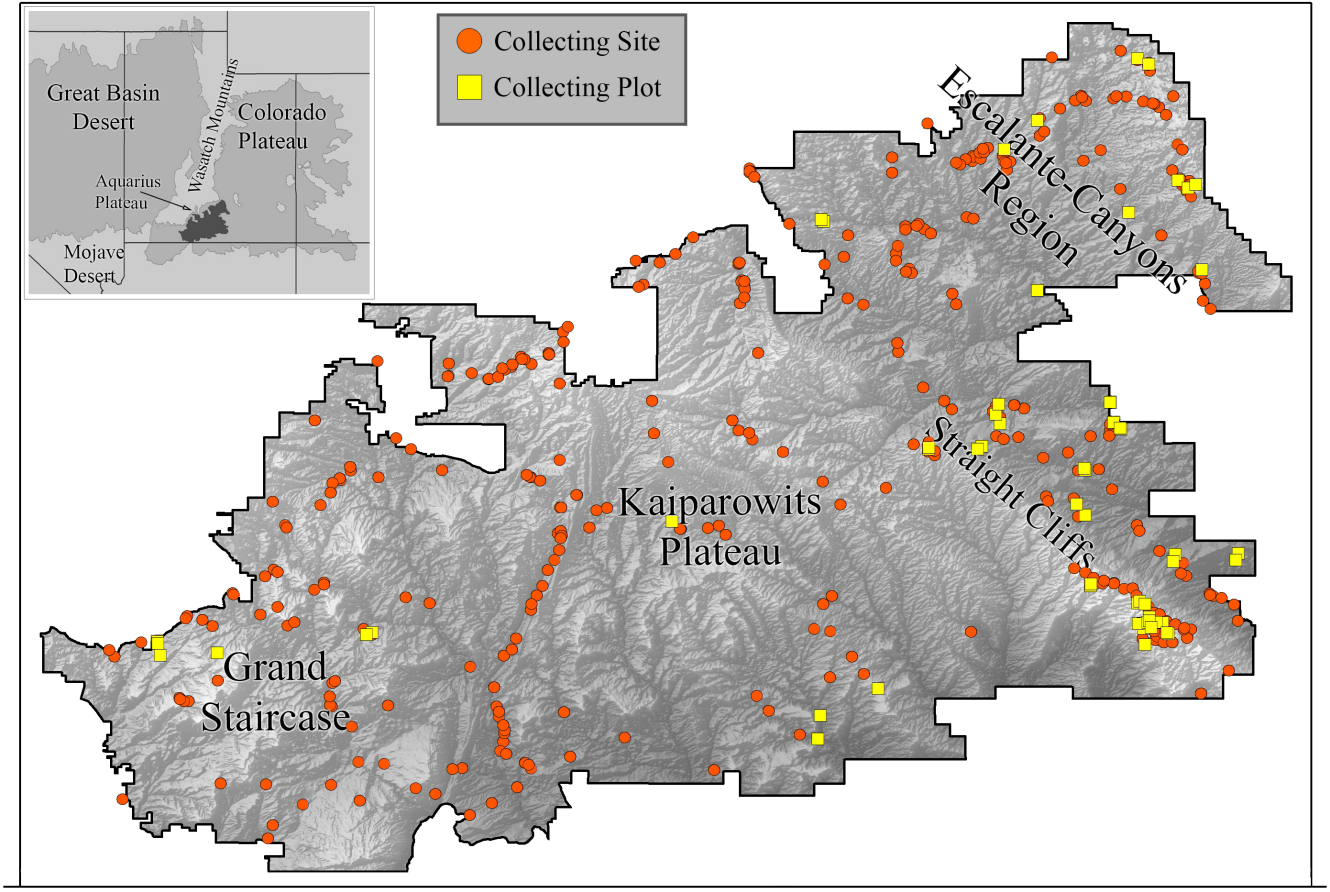
Collecting locations in the Grand Staircase-Escalante National Monument. Boundaries of the Grand Staircase-Escalante National Monument (from the years 2000–2003, when this study was conducted) are shown. Orange circles indicate areas where collections occurred. Yellow squares are plot locations.

We collected bees in GSENM over a period of four years (2000–2003) using both opportunistic collections and standardized bi-monthly sampling in one-hectare plots (Fig. 1). This follows a protocol commonly used for bee studies, providing the opportunity for comparisons with other areas, past and future (LeBuhn, 2003). Across GSENM, and across the four years of the study, a total of 66 plots, each 50 m × 200 m were established (Table 1); 11 plots were sampled all four years, 12 plots were sampled for three years, 12 were sampled two years, and 31 plots were sampled for just one year. Plot locations were stratified by habitat and represent a subset of plots documented by vegetation-mapping specialists working simultaneously in the monument (Stohlgren et al., 1998). Each plot was visited at approximately two-week intervals. On a given sampling day, pairs of collectors sampled each plot using aerial nets for two 45-minute periods—between 0900 and 1530, once before noon and once after. A plot visit, therefore, represents a total of 360 min (3 h) of collecting. An effort was made to collect the entire area of the plot evenly during a sampling period, and to collect from all bee-visited flowering plants in the plot. Collections were biased against honey bees—given a choice between collecting a feral honey bee, *Apis mellifera* Linnaeus and another bee, the other bee was collected. Attempts were made to collect every native bee encountered. Most plants on which bees were collected were identified to species using regional keys (Welsh et al., 1993). The remaining plants were identified to genus. The number of plots established for regular visitation was balanced against coverage across the monument. Those plots that were sampled for multiple years were chosen because of their accessibility and because they were representative of the majority of the monument’s habitats.

**Table 1:** Number of specimens and species collected per year, within and outside of plots for GSENM. A collector-day is any unique collector and date combination (i.e., a day that a collection was made by an individual). In total 80,859 specimens were collected across the four years.

Number of	2000	2001	2002	2003	Total
Collector-days	334	460	440	398	1,632
Start date	28 Apr	16 Apr	8 Apr	22 Apr	
End date	4 Oct	8 Oct	10 Oct	5 Oct	
Species	384	434	415	496	660
Specimens per collector day	81.75	45.44	56.33	47.23	49.55
Plots	43	41	30	17	66
Specimens in plots	13,844	10,958	11,201	5,178	41,181
Species in plots	307	369	308	291	514

To expand our understanding of plant-bee relationships and bee distributions, we also collected bees opportunistically throughout the monument in areas outside of plots, with the intention of sampling all accessible areas of the monument at least once in the spring and fall, especially targeting plants not present in plots. For clarity, subsequent use of ‘plot’ refers to systematically collected one-hectare plots, ‘site’ refers to any area in which collections were made outside of plots. Over the four years, 478 sites were sampled (Fig. 1).

Bees were pinned and labeled and are now housed in the US National Pollinating Insects Collection at the USDA-ARS Pollinating Insects Research Unit in Logan, Utah, with vouchers returned to GSENM. All specimens were identified to species using published keys (Adlakha, 1969; Alexander, 1994; Baker, 1975; Blair & Cockerell, 1935; Bohart, 1947; Bohart, 1948; Bohart, 1949; Bouseman & LaBerge, 1978; Broemeling, 1988; Brooks, 1983; Brooks & Griswold, 1988; Brumley, 1965; Daly, 1973; Danforth, 1994; Danforth, 1999; Donovan, 1977; Evans, 1972; Gibbs, 2010; Gonzalez & Griswold, 2007; Gonzalez & Griswold, 2013; Grigarick & Stange, 1968; Griswold & Parker, 1988; Hurd Jr, 1955; Hurd Jr, 1958; Hurd Jr & Linsley, 1951; Hurd Jr & Linsley, 1967; Hurd Jr & Linsley, 1972; Hurd Jr & Michener, 1955; LaBerge, 1956a; LaBerge, 1956b; LaBerge, 1957; LaBerge, 1958; LaBerge, 1961; LaBerge, 1967; LaBerge, 1969; LaBerge, 1971; LaBerge, 1973; LaBerge, 1977; LaBerge, 1980; LaBerge, 1985; LaBerge, 1987; LaBerge, 1989; LaBerge & Bouseman, 1970; LaBerge & Ribble, 1972; LaBerge & Ribble, 1975; LaBerge & Thorp, 2005; Linsley, 1939; McGinley, 1986; McGinley, 2003; Michener, 1938; Michener, 1939; Michener, 1947; Michener, 1949; Michener, 1968; Michener, McGinley & Danforth, 1994; Mitchell, 1934; Mitchell, 1935; Mitchell, 1936; Mitchell, 1937a; Mitchell, 1937b; Mitchell, 1937c; Mitchell, 1937d; Orr et al., 2016; Parker & Griswold, 2013; Ribble, 1968a; Ribble, 1968b; Ribble, 1974; Rightmyer, 2008; Rightmyer, Griswold & Arduser, 2010; Roberts, 1973; Rozen Jr, 1958; Rust, 1974; Rust & Bohart, 1986; Sandhouse, 1939; Snelling, 1966a; Snelling, 1966b; Snelling, 1966c; Snelling, 1966d; Snelling, 1967; Snelling, 1968; Snelling, 1970; Snelling, 1983; Snelling, 1990; Stage, 1966; Stephen, 1954; Stephen, 1957; Thorp, 1969; Thorp & LaBerge, 2005; Timberlake, 1954; Timberlake, 1956; Timberlake, 1958; Timberlake, 1960; Timberlake, 1962; Timberlake, 1964; Timberlake, 1968; Timberlake, 1969; Timberlake, 1971; Timberlake, 1973; Timberlake, 1975; Timberlake, 1976; Timberlake, 1980; White, 1952; Williams et al., 2008) and the extensive reference collection available in the Logan collection and were confirmed by the authors. Males of *Lasioglossum* (*Dialictus*) were not keyed to species (there is no revision for this subgenus and sex associations are not currently possible). This means that our species estimates for the monument are potentially conservative, if male specimens that are not identified include species not represented by identified females.

## Results

Over 80,000 bee specimens were collected across 1,632 collector-days. Of all specimens, 41.5% of individuals and 71.9% of species were collected in plots, and 58.5% of individuals and 95.8% of species were found in sites outside of plots.

### Diversity

**Figure 2:**
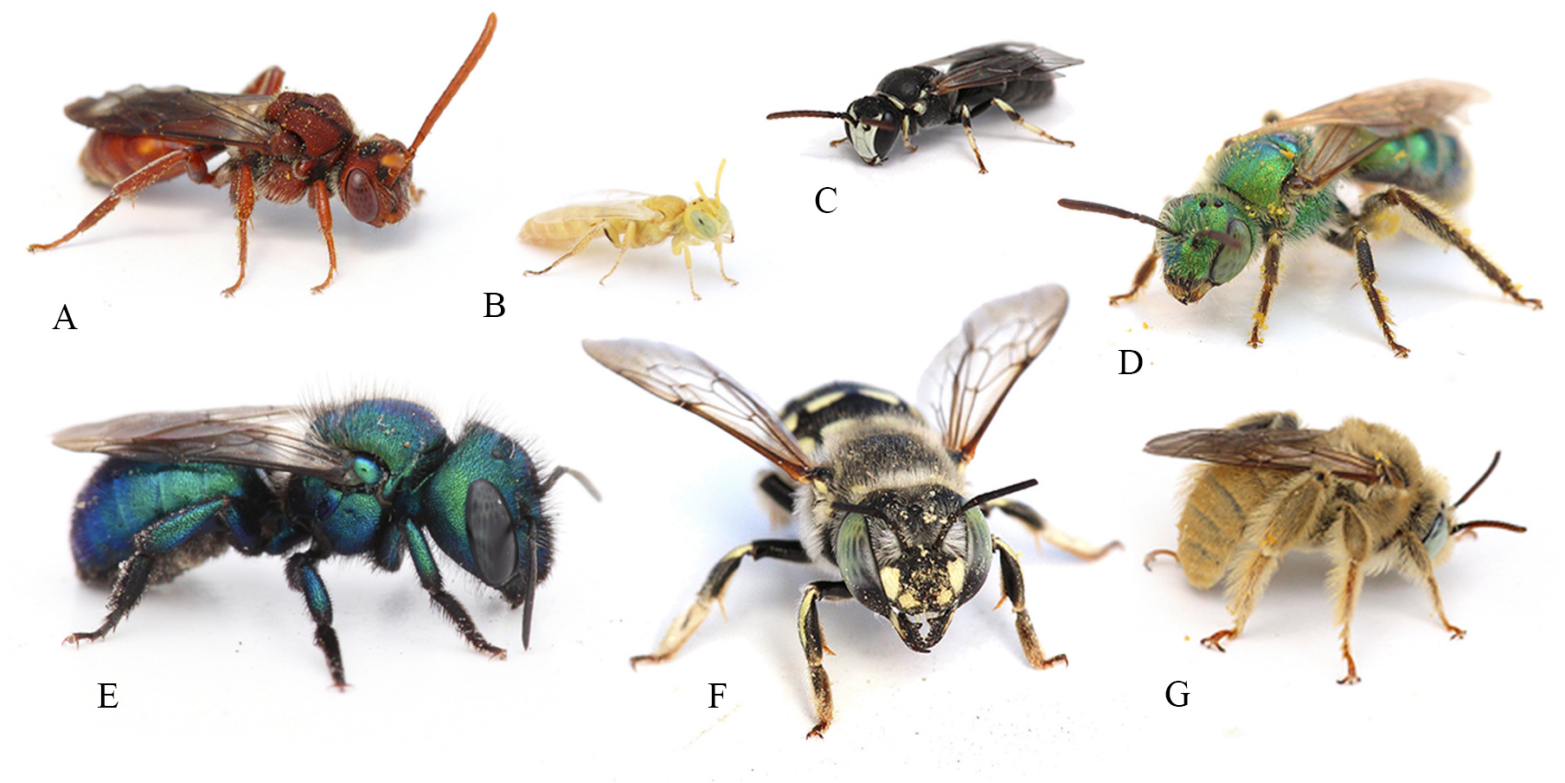
Examples of some of the bee genera found in Grand Staircase-Escalante National Monument. The following genera are pictured: (A) *Nomada*, (B) *Perdita*, (C) *Hylaeus*, (D) *Agapostemon*, (E) *Osmia*, (F) *Anthidium*, and (G) *Diadasia*. Photos by Joseph S Wilson.

GSENM encompasses a rich bee fauna (Fig. 2); 660 species in 55 genera (Table S1) were recorded from GSENM, including all six bee families extant in North America. This represents approximately 55% of the described bee species known from the state of Utah, and about 64% of bee species found on the Colorado Plateau (based on querying USDA ARS Pollinating Insect Collection’s extensive database of over 1.3 million records). Among GSENM’s bees are 49 undescribed species (previously unknown to science) (Table S1), and 13 species previously unrecorded from Utah (Table S1).

Several range extensions are documented through collections in GSENM. Thirteen species, including two cleptoparasitic genera (*Hexepeolus* and *Paranomada*) were previously unknown from Utah. An unnamed species of *Osmia* (*Melanosmia,* n. sp. 1) is a narrow endemic; we have yet to collect it outside the monument’s boundaries. An infusion of faunal elements from the hot deserts of the southwestern US and the cold deserts of the Great Basin can also be found in GSENM. Several collected species were previously thought to be restricted to the Mojave Desert (Table S1). Additionally, populations of multiple Great Plains disjuncts were discovered (Table S1).

Andrenidae is the most speciose family in GSENM (181 species), with most of those bees being either *Perdita* (49% of Andrenidae, and 87 species) or *Andrena* (39%, and 71 species). *Perdita* was also the most commonly collected genus accounting for 26.89% of all specimens collected*.* Apidae contains the most genera (39%, 22 genera). Within this family *Nomada*, a cleptoparasitic genus, is the best represented, with 34 species. Halictidae are dominated by *Lasioglossum* species, especially *Lasioglossum* (*Dialictus*) (9.92% of all specimens collected comprising 58 species)*.* Finally, *Hylaeus* (Colletidae) was the third most commonly collected genus (6.11% of all specimens, 12 species).

The majority of bees in GSENM are solitary, though several social species (species with at least some populations that show a reproductive division of labor) also occur, including eight species of *Bombus*, five *Halictus* species, nearly 70 *Lasioglossum* species (most *Lasioglossum* (*Dialictus*) species are thought to be social but the sociality of most species has not been investigated), and the non-native honeybee, *Apis mellifera*. While these social species represent just over 1% of the overall fauna, they account for 16.8% of collected specimens.

Cleptoparasites accounted for 18% of all species. Excluding cleptoparasites, a majority (71%) of bees in GSENM are known to be ground nesters (bees that excavate tunnels in the earth, in which they lay eggs and leave pollen provisions). Limited numbers nest in stems (6%) or wood (6%) with the remaining 17% either having other nesting habits (i.e., in sandstone or in external resin nests) or their nesting habits are unknown.

### Spatial patterns

While most bee species from GSENM were neither abundant nor widespread, there were a handful of species that dominated. Seven species (1% of the fauna) accounted for nearly 29% of all collected specimens, based on all collections for the monument. Four of these were *Perdita*: *P. calloleuca* Cockerell (7%), *P. subfasciata* Cockerell (4%), *P. zebrata flavens* Timberlake (3%), and *P. similis* Timberlake (2%). *Ceratina nanula* Cockerell (6%), and *Halictus tripartitus* Cockerell (4%) were also well represented. Finally, *Apis mellifera* accounted for three percent of all specimens collected. Though these seven species were extremely abundant overall, and consistently occurred in large numbers at any location, no bee was ubiquitous. *Perdita calloleuca*, the most abundant bee, in its year of greatest abundance (2003), was found in only 42 of 136 (30%) sites collected in the fall (when it is active), and in 10 of 14 systematically collected plots.

**Figure 3:**
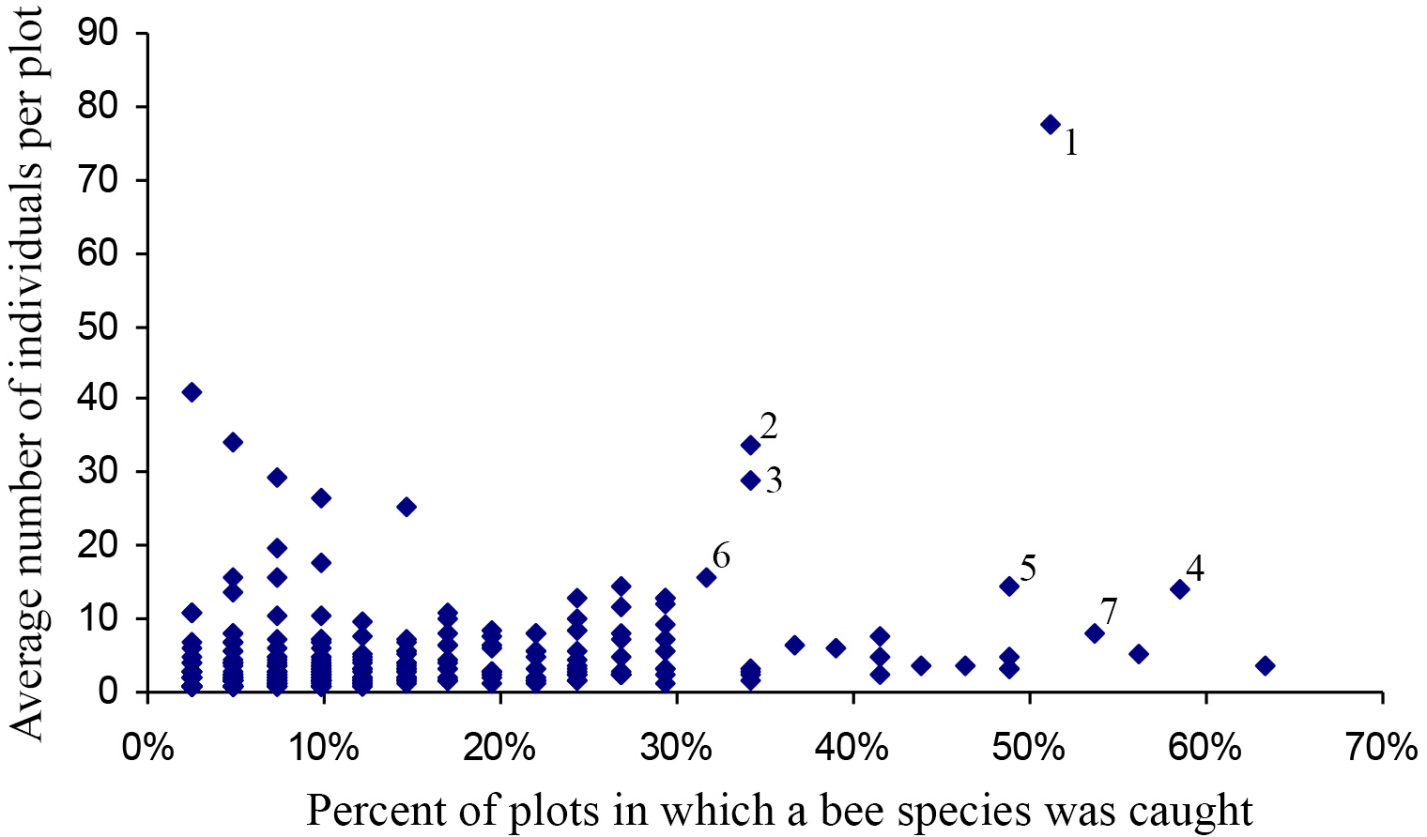
Number of plots in which a species was found versus the average number of individuals when present. Species towards the upper right are widespread and abundant, and are numbered as follows: (1) *Halictus tripartitus* (2) *Perdita subfasciata* (3) *Perdita calloleuca* (4) *Apis mellifera* (5) *Ceratina nanula* (6) *Andrena lupinorum* (7) *Perdita aridella*. Species high on the graph and towards the right are widespread and abundant. Species high on the graph and towards the left are localized and abundant. The rarest species are in the lower left, and species that are widespread but never abundant are to the right and low. Species high on the graph and towards the left are localized and abundant. The rarest bees are in the lower left, and species that are widespread, but never abundant, are to the right, and low.

**Figure 4:**
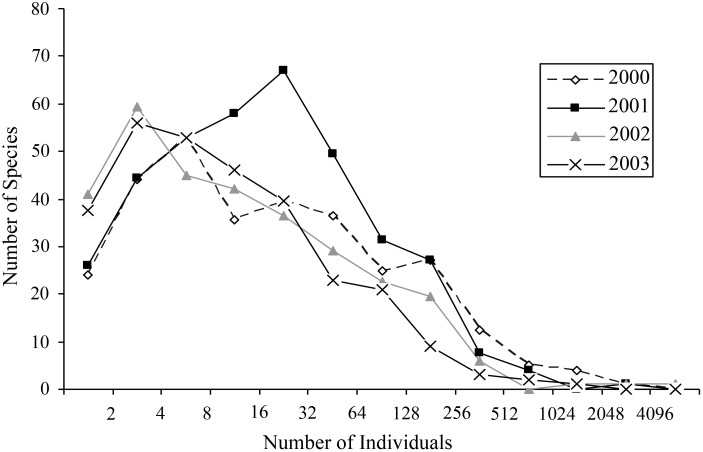
Octaves of bee abundance in plots. Octaves of abundance for species found in plots for each of the four years in GSENM. Values on *x*-axis represent *n* in the equation *y* = 2^*n*^.

Most species occurred in very low numbers and were found in few plots (Fig. 3). On average, 4.9 individuals of any species were collected per plot. While over four years a mean of 149.65 individuals were collected of each species, the median was 27 individuals (SD = 463.78), indicative of the highly skewed rank-abundance (Fig. 4). Seventeen percent of bees are singletons or doubletons, rarely occurring in the monument. Alternatively, some species were abundant but extremely localized (Fig. 3). *Perdita festiva* Timberlake was collected in only seven sites, but averaged 171 individuals per site and was in fact the eleventh most commonly collected bee. It is a specialist on Asteraceae, and appears to favor *Ericameria nauseosus,* which is widespread throughout the monument. Two new species, *Hylaeus* n. sp. aff*. mesillae* and *Perdita* n. sp. aff*. confusa* were both found in only two sites, but were very abundant when found, with over 75 individuals collected at each locality.

Within the monument, certain regions harbor distinct faunas. High elevation sites included isolated populations of species not found elsewhere in the monument, but present outside the monument at similarly high elevations. Twelve species collected at higher elevations on the Aquarius Plateau, north of the monument (Fig. 1), were restricted to the adjacent northern edge of GSENM. An additional 44 species were also found only along the northern boundary of the monument (within 10 km of the border); however, records of these species on the Aquarius Plateau do not exist for comparison. The Straight Cliffs, which divide the Escalante-Canyons region from the Kaiparowits Plateau (Fig. 1), appear to limit distributions of some bee species. *Protosmia rubifloris* Cockerell, a primarily cismontane California species, extends to the eastern edge of the Kaiparowits Plateau, but not beyond. A number of species were present only at lower elevations in the Escalante-Canyons Region, or, if found further west, were found only at low elevations along the monument’s southern border. These bees include: *Agapostemon splendens* (Lepeletier), *Andrena* n. sp. *(*aff. *pecosana), Andrena cressonii* Robertson*, Andrena papagorum* Viereck & Cockerell*, Dianthidium arizonicum* Rohwer*, Eucera lunata* (Timberlake), *Megachile townsendiana* Cockerell, and * Pseudopanurgus irregularis* (Cockerell).

**Figure 5:**
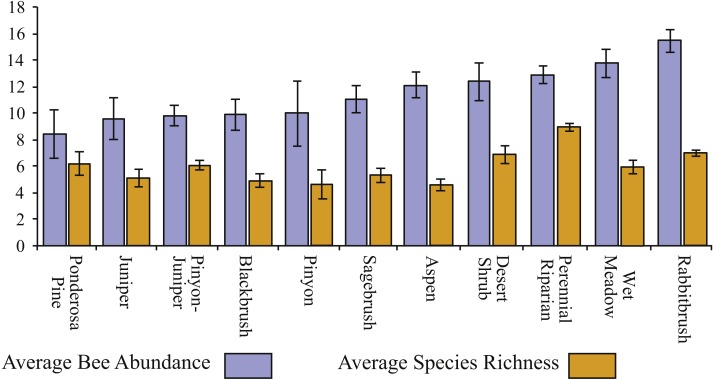
Bee richness and abundance by habitat. Average bee species abundance for each habitat collected in GSENM is represented with blue. Average bee richness for each habitat is represented with orange. Bars represent 1 standard deviation.

Bee richness and abundance both differed depending on habitat. While *Chrysothamnus* (sensu lato) dominated landscapes resulted in the greatest number of individuals collected (on average), it was perennial riparian areas, with year-round water that resulted in the greatest species richness (Fig. 5). Ponderosa pine, juniper, and pinyon-juniper habitats seldom resulted in many individuals, while aspen, pinyon, and blackbrush (*Coleogyne ramossisima*) areas harbored the fewest number of species.

### Seasonal patterns and floral relationships

**Figure 6:**
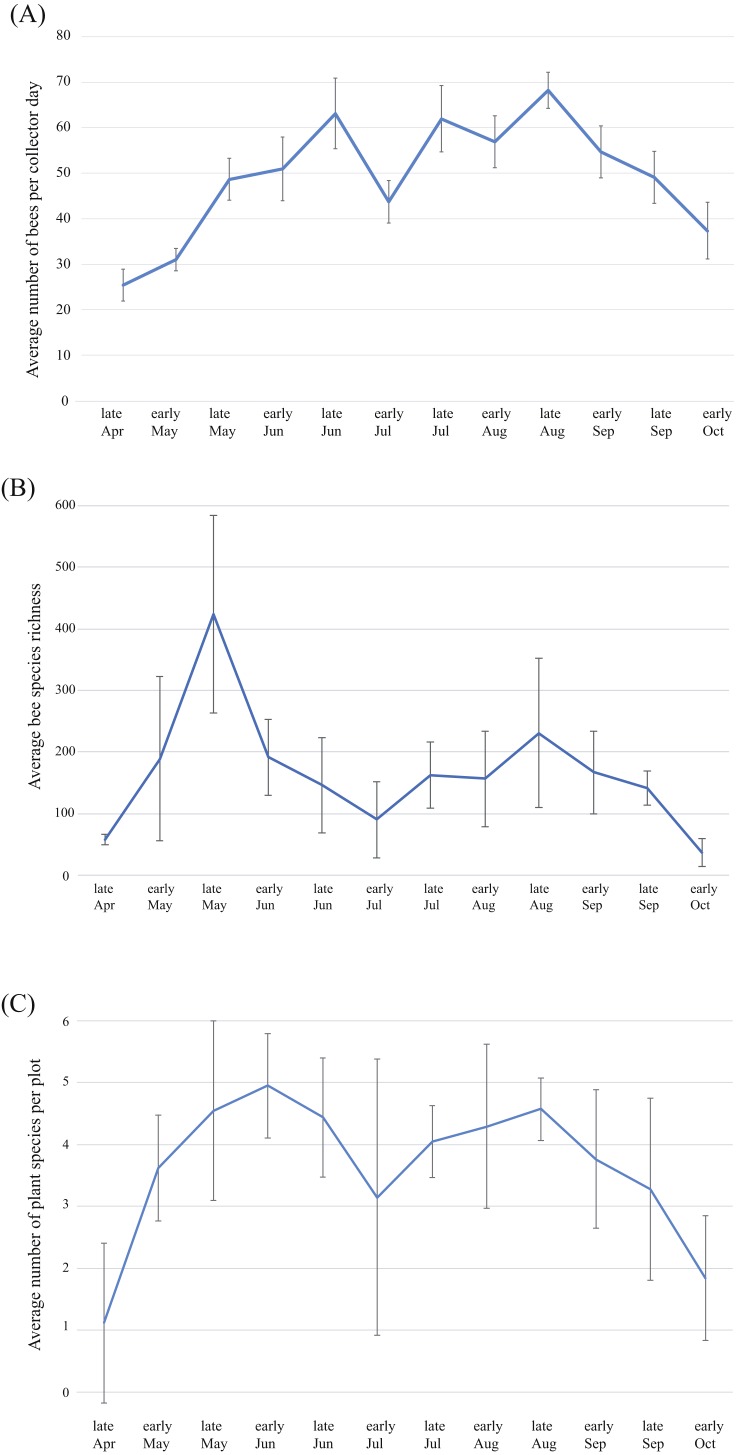
Patterns of bee and plant richness and abundance across the year. (A) Average bee species abundance per collector-day across the flowering season, (B) total bee species richness for each half-month of collections, for the four years of the study and (C) average number of plant species in flower for each half-month of collections across the flowering season. Bars represent 1 standard deviation. Note that bee abundance peaks in late June and late August, though diversity does not increase at the same time. This is due to social species, which become abundant during the summer months.

Average bee abundance follows a bimodal pattern across the flowering season, with a higher peak in mid-August compared to the spring (Fig. 6A). Species richness follows a similar pattern though with slightly higher species richness in the spring compared to the summer fauna (Fig. 6B). In each of the four years surveyed, the greatest species richness was documented in the spring (early to late May), followed by a second smaller but broader late summer peak in the fall months (early August to early September). Species richness across the season roughly mirrors precipitation that accompanies the monsoon season and accumulated winter precipitation in the spring. Peaks in floral diversity in plots are similar to the peaks in bee diversity and abundance (Fig. 6C).

Bees were collected from over 375 flowering species of the more than 1,000 present in GSENM (Fertig, 2005). Floral specialization, or oligolecty, is typically found at the generic level (i.e., a specialist bee species forages primarily on a specific genus of flowering plants rather than on a specific flowering species) (Cane & Sipes, 2006); hence our summary of floral relationships focuses at the generic level. We collected from 161 flowering plant genera in our four years in GSENM.

Seventeen plant genera hosted 75% of all bee specimens collected in plots (Table 2). Flowers may be host to a high number of bee species or individuals because they are widespread or bloom for a long period of time. In total, 13,745 (6,490 in plots) specimens were collected on *Chrysothamnus* (sensu lato) and it was present in 138 (32.09%) of all locations, including 37 plots. It also bloomed for five consecutive months of the year. However, some other plants that were very attractive were found in few plots (e.g., *Salix* and *Thelypodium*), or were found only in sites rather than plots (e.g., *Psoralea,* which hosted 97 bee species and 1,346 individuals).

Plants that attracted the greatest number of individuals were not the same plants that attracted the greatest number of species (Table 2). *Cryptantha*, which attracted a relatively low 2,000 specimens (2.47%), accumulated 184 species. *Lupinus,* which was found at higher-elevations, hosted 107 species, and only 836 individuals. Alternatively, *Salsola,* which attracted 3.54% of all specimens in plots attracted only 70 species.

**Table 2:** Attributes of Magnet Plants ordered by overall visitors. These plant genera hosted 75% of all specimens collected in plots.

**Family**	**Genus**	**Plots**	**Ad**	**Species**	**a/p**	**FL**	**N**		**R**	
							Plots	Sites	Plots	Sites
Asteraceae	*Chrysothamnus* (sensu lato)	37		7	p	Jul–Nov	6,490	13,745	180	236
Tamaricaceae	*Tamarix*	15	1/1 sp.	1	p	May–Sep	5,559	10,175	166	218
Asteraceae	*Senecio*	30		6	p	May–Sep	2,257	3,110	119	169
Chenopodiaceae	*Salsola*	14	2/2 sp.	2	a/p	Jul–Sep	2,249	2,970	52	70
Fabaceae	*Melilotus*	10	1/2 sp.	2	a	May–Oct	1,941	4,386	110	160
Asteraceae	*Erigeron*	33		19	a/p	May–Sep	1,475	1,810	119	141
Asteraceae	*Gutierrezia*	34		2	p	Jul–Oct	1,419	2,648	101	138
Capparidaceae	*Cleome*	23		2	a	May–Sep	918	4,058	95	169
Malvaceae	*Sphaeralcea*	27		3	p	May–Sep	914	2,790	103	166
Asteraceae	*Taraxacum*	17	1/1 sp.	1	p	Jan–Nov	903	1,334	82	102
Asteraceae	*Cirsium*	12	1/8 sp.	8	p	May–Sep	798	1,195	59	90
Asteraceae	*Heterotheca*	13		2	p	May–Oct	789	1,143	88	110
Polygonaceae	*Eriogonum*	28		23	a/p	May–Sep	592	2,065	87	130
Lamiaceae	*Poliomintha*	14		1	p	Apr–Jun	510	1,219	68	120
Salicaceae	*Salix*	6		10	p	Apr–Jul	508	1,272	54	110
Brassicaceae	*Thelypodium*	4		1	p	Aug–Oct	513	774	59	77
Boraginaceae	*Cryptantha*	36		20	a/p	May–Jul	476	1,996	98	184

**Notes.**

Plots Number of plots in which genus occurred Ad Adventive (number of adventive species/total members of the genus in GSENM) Species Number of species in the plant genus in GSENM (according to  Welsh & Atwood, 2002) a/p Annual or Perennial FL Flowering Time R Bee richness in plots or sites N Number of bee individuals in plots or sites

Magnet plants, those that attract an extraordinarily diverse bee fauna, share few life-history traits. Several of these plants are annuals while others are perennial. Both spring and fall bloomers are represented, as are genera that span the seasons, most notably the invasive and widespread *Tamarix*—a large shrub/tree that can be found blooming from May through August. Both invasive and native plants supported bees: *Chrysothamnus* (sensu lato) hosted the most individuals and was also the most widely represented within plots. In contrast, several of the plants supporting the greatest abundance of bees are invasive, with *Salsola* and *Tamarix* both attracting a large number of specimens. Interestingly, neither plant attracted a large number of species compared to the number of specimens collected.

Despite the obvious attraction of a relatively few key plants within GSENM, bees exhibit a broad range of floral preferences. Social species are obligate generalists, but many solitary bees are equally catholic in their floral affinities. At the other extreme, numerous species in GSENM are oligolectic. One hundred eighty-two of the 552 species in GSENM that are not parasites (33%) are documented specialists (Hurd Jr, 1979). This is a conservative estimate; the floral preferences of many bees remain unclear, so the true number of specialists may be significantly higher.

Oligoleges, despite their narrower plant preferences, are not in general more localized, nor less abundant than polyleges (floral generalists) in GSENM. Three of the most common and widespread bees in the monument are oligolectic, two on tribes within Asteraceae (*Perdita subfasciata* and *Perdita similis*), and one (*Perdita zebrata flavens*) on *Cleome* spp. Indeed, documented oligoleges were collected at an average of 23.8 sites, whereas polyleges and those with unknown floral preferences (excluding parasites) were caught at an average of 20.7 sites.

### Interannual variation

The twelve plots that were sampled systematically across all four years yielded 388 species. Of those, 136 were collected in just one year (35%), and 83 (21%) were collected in only two of the four years. In these twelve plots, 99 species (26%) were collected in all four years of the study, but the number of individuals collected for each of these species varied greatly between years, and the variation did not follow a consistent upward or downward trend. Different species reached peak abundance in each of the four years. For example, 189 specimens of *Agapostemon texanus/angelicus* were collected in 2000, 32 in 2001, 50 in 2002, and 10 in 2003. In contrast, 45 specimens of *Anthophora urbana* were collected in 2000, 40 in 2001, 166 in 2002, and 36 in 2003. Species that were abundant in a given year were not widespread across the monument in that same year. Moreover, bees that were widespread reached peak abundance at different locations in different years. As an example, *Bombus morrisoni* was most abundant at high elevation sites in 2000, but most abundant along a low elevation riparian area in 2002. *Ceratina nanula* population levels changed by an order of magnitude between years (203, 11, 142, and 11, respectively) at just one plot.

## Discussion

The research presented here enumerates and describes the bee community of GSENM. Our intensive, long-term study reveals an extraordinarily diverse, unique, and dynamic bee population. The majority of the species were not abundant, were spatially localized, and experienced significant interannual fluctuations in bee population size. If GSENM is indicative of other regions of the Colorado Plateau, future surveys in the ecoregion will likely also record a rich and distinct fauna.

### Diversity

The bee richness of GSENM compares favorably with other well-sampled regions of North America (Michener, 1979; Williams, Minckley & Silveira, 2001, Table 3). Despite the relatively small geographic area, the number of bee species found in GSENM is comparable to the number of bee species known from the entire eastern US (everything east of the Mississippi River: Colla et al., 2012; Ascher & Pickering, 2018).

**Table 3:** Published records of bee faunas for various parts of North America, ranked by number of species.

**Location**	**Area (**Km**^**2**^)**	**Species**	**Genera**
All U.S. areas east of the Mississippi River (Colla et al., 2012)	2,424,045	770	43
Grand Staircase-Escalante Nat’l Mon.	7,689	660	55
Clark Co. Nevada (Griswold et al., 1999)	20,878	598	67
Pinnacles Nat’l Mon. (Messinger & Griswold, 2002)	65	398	52
Curlew Valley, UT & ID (Bohart & Knowlton, 1973)	592	340	43
San Rafael Desert (Griswold, Parker & Tepedino, 1998)	7,744	333	49
Albany Co., WY (Tepedino & Stanton, 1981)	11,160	194	40
San Mateo County, CA (Moldenke, 1976)	1,930	163	
Okanagan Nat’l Forest (Wilson et al., 2010)	6,066	140	24
Everglades National Park (Pascarella, Waddington & Neal, 1999)	6,110	104	34

GSENM’s vast size (it covers 3% of Utah’s land area), the wide range of elevations and diverse geologic profile it includes, and the resulting assortment of habitats and soil types likely are at the root of the diversity of bees found here. In addition to a wide array of nesting substrates and materials, these landscape variables allow for a rich flora (Fertig, 2005), with a variety of bee-attractive plant families and genera (Table 1). Conservatively, 33% of bee species in GSENM are known floral specialists. Such diversity provides a wide selection of floral niches and could potentially minimize pollen and nectar limitation. Additionally, the short lifespan of many bee species within GSENM (less than nine weeks), compared to the longer duration of many perennial plants, and bees’ modest distributions compared to that of many of the plants could have facilitated the development of multiple temporally isolated bee communities all associated with the broader (both geographically and temporally) plant communities.

GSENM encompasses a region of Utah that includes both hot and cold desert elements (Fig. 1), which may have acted as refugia throughout the Pleistocene due to the regions climatic stability through interglacial and glacial cycles (Wilson & Pitts, 2012). To the north and west of GSENM is the Great Basin Desert, and GSENM sits squarely on the Colorado Plateau. Both the Colorado Plateau and the Great Basin are considered cold deserts; they experience many frost-days each year, and a significant portion of annual precipitation falls as snow. As a result, the flora of GSENM is similar to the frost-hardy flora of the Great Basin (Cronquist et al., 1972; Harper et al., 1978), though there are some differences: the flora of the Colorado Plateau includes more summer-growing plants, and a greater diversity in plant structure, physiology, and growing period (Comstock & Ehleringer, 1992). Many of the plant species that are host to specialist bees in the Great Basin are also found in GSENM. The plant genera most attractive to bees in the Great Basin (Bohart & Knowlton, 1973; Toler, 2001) are very similar to those plants most attractive to GSENM bees.

It is surprising then, that there are few cold desert-adapted bee species from the Great Basin present in GSENM. Almost all species that occur in both GSENM and the Great Basin are widespread throughout the West, including areas outside the Great Basin. In contrast, a distinctive Mojavean element, associated only with that hot desert, is evidenced by the presence of several bee species from the warm deserts to the south. This suggests that, while floral conditions in GSENM should be hospitable to Great Basin bees (and vice versa), there is some other factor limiting the exchange of species between these two regions. One possibility is the southern tail of the Wasatch Mountains (Fig. 1), which may be an insurmountable physiographic barrier for many bees between the Colorado Plateau and the Great Basin. Such a barrier is less evident between the Colorado Plateau and the Mojave Desert, where the Colorado River drainage may serve as a hospitable conduit for bee species from the Mojave Desert. Studies of the Vermilion Cliffs and Grand Canyon regions south of the GSENM may shed light on this.

### Floral relationships

Several plant species were overwhelmingly attractive and may exert inordinate influence on bee diversity, though the nuances of how floral composition correlate to bee population dynamics remain unclear. In GSENM, magnet plant species differ in many attributes, but are similar in that they are all predictable resources, and are mostly perennial. Regardless of rainfall, they bloom consistently from year to year, bloom in abundance, and/or are widespread.

Previous investigations into the interaction of bees and flowering resources show contradictory responses of bees to the presence and abundance of flowering plants (Denning & Foster, 2017; Potts et al., 2003; Tepedino & Stanton, 1981). For example, remnant prairies and reconstructed prairies in Kansas differed in the composition and abundance of flowering plants (Denning & Foster, 2017) but showed no significant difference in richness or abundance of bees. In contrast, Tepedino & Stanton (1981) found constant and significant positive correlations between floral richness and bee richness at prairie sites in Wyoming. The differing responses of bees to floral richness may be explained in part by the traits of a few dominant plants that provide pollen and nectar to a large array of bee species (e.g., Warzecha et al., 2017). Specifically, the presence of perennial, predictable plants may indicate a landscape with a diverse bee population, but those same plants may not correlate well with bee richness at a smaller scale. Thus, when Potts et al. (2003) divided plants in his study sites according to this life history trait, annual plants were better correlates of bee diversity than perennials—i.e., populations fluctuate in accordance with unpredictable bloom, not with those that are consistent. Likely the two plant habits (perennial and widespread vs. annual and locally abundant) work in concert to support the greatest diversity. Finally, because bees are central-place foragers, the presence of perennial, predictable resources, may encourage them to nest in the same area for multiple years, so that bees in areas where magnet plants are present are not only more diverse, but also more consistent between years (Venturini et al., 2017; Scheper et al., 2015), but see also (Franzen & Nilsson, 2013), perhaps explaining the high richness in the *Chyrsothamnus-*dominated plots.

It is interesting to note the number of bee-attractive plants in the monument that are exotic. Because the pollen loads of bees collected on these plants have not bee examined yet, it is unknown whether they were visiting for nectar rewards or were opportunistically provisioning nest cells with exotic pollens. Their effectiveness at pollinating these invasives is also unknown. Nonetheless, there is evidence from other ecosystems to suggest that invasives affect plant–pollinator networks, and this likely holds true for GSENM (McKinney & Goodell, 2010; McKinney & Goodell, 2011; Tepedino, Bradley & Griswold, 2008; Vanbergen, Espíndola & Aizen, 2018; but see Charlebois & Sargent, 2017), especially considering that many of the most commonly visited plants in the monument are invasive.

### Spatial patterns and conservation implications

Williams, Minckley & Silveira (2001), in a review of existing faunal surveys, noted that studies of patterns of bee abundance, of any duration or intensity, include many poorly represented species and only a few that are super-abundant. More recent studies have similarly found beta-diversity to be high in bees (e.g., Dorchin et al., 2017), with considerable turnover across a landscape and many localized species. With bees, rarity may be a persistent property; in GSENM few species were abundant, and there were typically 40–50 species per plot (out of a possible 475 collected in plots). The implications of this variability are significant; prioritizing areas for conservation is complicated when each area represents a distinct community (Rodrigues et al., 2004). Setting aside large interconnected areas may be especially important for maintaining bee communities, both because of the localized nature of all but a few species, and also because the floral resources on which they rely must be considered within a connected landscape matrix, rather than individually. This is further complicated by the varying life-history traits even among the dominant bees, suggesting that a multitude of resources might be needed to maintain bee communities (Everaars, Settele & Dormann, 2018; Winfree et al., 2018; Harrison, Gibbs & Winfree, 2017; Nicholson et al., 2017; Heard et al., 2007; Cane et al., 2006).

### Interannual variation

Bees in GSENM vary substantially from year to year in terms of their abundance. This pattern has been observed in other areas as well (reviewed by Williams, Minckley & Silveira, 2001, also Tepedino & Stanton, 1981; Kimoto et al., 2012), though the drivers are not well-understood. A one-year lag with floral resource abundance has been observed in other areas (Tepedino & Stanton, 1981; Potts et al., 2003; Inari et al., 2012; Crone, 2013) but has not been evaluated for desert environments like GSENM. Considering that not all bees fluctuated in the same direction, or in the same years, it seems possible that bee guilds experience floral resource fluctuations, parasite loads, and disease dynamics differently. Recognizing that bee populations can vary dramatically from year to year, as we found in this study, is important for bee researchers who are investigating the presence, or extent, of native bee declines (Cane & Tepedino, 2001; Lebuhn et al., 2013).

## Conclusions

Our study of bees from GSENM represents, to date, one of the largest published bee surveys both in terms of geographic area covered and consecutive years sampled. This long-term study not only reveals GSENM to be a hot spot of bee biodiversity, an area previously not well-known in terms of its bees, it is also evidence of high spatial heterogeneity in arid-adapted bee communities. It adds to our knowledge of bee-plant relationships and the role of ‘magnet plants’ in maintaining a bee fauna between years. Finally, it highlights a bee fauna that naturally varies significantly from year to year, with populations of different species increasing and decreasing interannually with no clear trend. The protection of large areas that incorporate a variety of habitat types may be important, considering the spatial and annual heterogeneity of this, and likely other, desert bee faunas. Our data, and similar studies, also provide baseline data, which are pivotal in the continued investigations of bee declines.

##  Supplemental Information

10.7717/peerj.5867/supp-1 Supplemental Information 1 A list of all identified bee specimens collected in Grand Staircase Escalante National Monument between 2000 and 2003 Bees are arranged by family, subfamily, genus, subgenus, and finally species. New species (n. sp.) are those that differ from all published keys and are unique after comparison with all specimens located at the U.S. National Pollinating Insects Laboratory (a collection of nearly 2 million specimens). They are listed with their closest affiliation. Species that do not match with specimen descriptions exactly, nor with known specimens, but are not entirely distinct may represent either variants or new species. We have conservatively listed these as ‘sp.’, rather than as new species, with the closest affiliation in parentheses. Notes of biogeographical interest are in the last column, and are coded as follows: CP: Endemic to the Colorado Plateau; E: Easternmost occurrence of the bee; GB: Endemic to the Great Basin; GSENM: Found only in GSENM; MD: Previously recorded only in the Mojave Desert; NG: New genus for the state of Utah; N: Northernmost occurrence of the bee; NS: New species for the state of Utah; SD: Previously reported only in the Sonoran Desert; S: Southernmost occurrence of the bee; W: Westernmost occurrence of the bee. *Agapostemon angelicus and A. texanus females are impossible to distinguish. The number of specimens reported for each of these two species is for the males only, which can be identified.

10.7717/peerj.5867/supp-2 Supplemental Information 2 Specimen data for all bees from the Grand Staircase-Escalante National Monument Data include specimen information, collection location information, and information about the floral hosts.

## Additional Information and Declarations

### Data Availability

The following information was supplied regarding data availability: The raw data are provided in the Supplemental Files.
