# Supplementary material for: Wild bees of Grand Staircase-Escalante National Monument: richness, abundance, and spatio-temporal beta-diversity
Source: PeerJ. 2018 Nov 7;6:e5867. doi: 10.7717/peerj.5867 (PMC6230437; doi:10.7717/peerj.5867)
Supplement: Supplemental Information 1 — Bees are arranged by family, subfamily, genus, subgenus, and finally species. New species (n. sp.) are those that differ from all published keys and are unique after comparison with all specimens located at the U.S. National Pollinating Insects Laboratory (a collection of nearly 2 million specimens). They are listed with their closest affiliation. Species that do not match with specimen descriptions exactly, nor with known specimens, but are not entirely distinct may represent either variants or new species. We have conservatively listed these as ‘sp.’, rather than as new species, with the closest affiliation in parentheses. Notes of biogeographical interest are in the last column, and are coded as follows: CP: Endemic to the Colorado Plateau; E: Easternmost occurrence of the bee; GB: Endemic to the Great Basin; GSENM: Found only in GSENM; MD: Previously recorded only in the Mojave Desert; NG: New genus for the state of Utah; N: Northernmost occurrence of the bee; NS: New species for the state of Utah; SD: Previously reported only in the Sonoran Desert; S: Southernmost occurrence of the bee; W: Westernmost occurrence of the bee. *Agapostemon angelicus and A. texanus females are impossible to distinguish. The number of specimens reported for each of these two species is for the males only, which can be identified. [file peerj-06-5867-s001.pdf]

Supplement A.

A list of all identified bee specimens collected in Grand Staircase Escalante National Monument between 2000 and 2003. Bees are arranged by family, subfamily, genus, subgenus, and finally species. New species (n. sp.) are those that differ from all published keys and are unique after comparison with all specimens located at the U.S. National Pollinating Insects Laboratory (a collection of nearly 2 million specimens). They are listed with their closest affiliation. Species that do not match with specimen descriptions exactly, nor with known specimens, but are not entirely distinct may represent either variants or new species. We have conservatively listed these as 'sp.', rather than as new species, with the closest affiliation in parentheses.

Notes of biogeographical interest are in the last column, and are coded as follows: CP: Endemic to the Colorado Plateau; E: Easternmost occurrence of the bee; GB: Endemic to the Great Basin; GSENM: Found only in GSENM; MD: Previously recorded only in the Mojave Desert; NG: New genus for the state of Utah; N: Northernmost occurrence of the bee; NS: New species for the state of Utah; SD: Previously reported only in the Sonoran Desert; S: Southernmost occurrence of the bee; W: Westernmost occurrence of the bee.

* *Agapostemon angelicus and A. texanus* females are impossible to distinguish. The number of specimens reported for each of these two species is for the males only, which can be identified.

Bee Species in GSENM

660 species, 55 genera, 49 new species

**Andrenidae, 181 species, 7 genera, 22 new species**		
Andreninae		
Ancylandrena, 1 species		
Ancylandrena timberlakei Zavortink, 1974	1	
Andrena, 71 species, 3 new species		
Andrena (*Andrena*) *frigida* Smith, 1853	7	
Andrena (*Andrena*) *macoupinensis* Robertson, 1900	26	
Andrena (*Andrena*) *milwaukeensis* Graenicher, 1903	339	
Andrena (*Andrena*) *schuhi* LaBerge, 1980	289	
Andrena (*Belandrena*) *sphaeralceae* Linsley, 1939	14	
Andrena (*Callandrena*) *accepta* Viereck, 1916	6	
Andrena (*Callandrena*) *haynesi* Viereck and Cockerell, 1914	3	W
Andrena (*Callandrena*) *helianthi* Robertson, 1891	5	
Andrena (*Callandrena*) *neomexicana* LaBerge, 1967	10	CP
Andrena (*Callandrena*) *pecosana* Cockerell, 1913	14	
Andrena (*Callandrena*) *utahensis* LaBerge, 1967	112	
Andrena (*Callandrena*) *vulpicolor* Cockerell, 1897	25	
Andrena (*Callandrena*) n. sp. 1	25	
Andrena (*Callandrena*) sp. 2 (aff. *simulata*)	1	
Andrena (*Callandrena*) sp. 1 (aff. *pecosana*)	8	
Andrena (*Cnemidandrena*) *ramaleyi* Cockerell, 1931	107	
Andrena (*Cnemidandrena*) *costillensis* Viereck & Cockerell, 1914	66	
Andrena (*Cnemidandrena*) sp. 3 (aff. *aurihirta*)	91	
Andrena (*Cnemidandrena*) sp. 4 (aff. *colletina*)	63	
Andrena (*Cnemidandrena*) n. sp. 2 (aff. *costillensis*)	21	
Andrena (*Conandrena*) *cheyennorum* Viereck & Cockerell, 1914	84	
Andrena (*Dactylandrena*) *porterae* Cockerell, 1900	2	
Andrena (*Dasyandrena*) *cristata* Viereck, 1917	5	
Andrena (*Diandrena*) *evoluta* Linsley & MacSwain, 1961	334	
Andrena (*Euandrena*) *astragali* Viereck & Cockerell, 1914	18	S
Andrena (*Euandrena*) *chlorura* Cockerell, 1916	23	
Andrena (*Euandrena*) *lawrencei* Viereck & Cockerell, 1914	11	
Andrena (*Euandrena*) *nigrihirta* (Ashmead, 1890)	13	
Andrena (*Euandrena*) sp. 5 (aff. *auricoma*)	1	
Andrena (*Euandrena*) sp. 6 (aff. *nigrihirta*)	3	
Andrena (*Holandrena*) *cressonii infasciata* Lanham, 1949	48	
Andrena (*Leucandrena*) *barbilabris* (Kirby, 1802)	17	
Andrena (*Melandrena*) *cerasifolii* Cockerell, 1896	44	

Andrena (*Melandrena*) *lupinorum* Cockerell, 1906	1082	
Andrena (*Melandrena*) *sola* Viereck, 1917	7	
Andrena (*Melandrena*) *transnigra* Viereck, 1904	28	
Andrena (*Micrandrena*) *illinoiensis* Robertson, 1891	7	
Andrena (*Micrandrena*) *kristina* Lanham, 1983	21	NS, W
Andrena (*Micrandrena*) *melanochroa* Cockerell, 1898	2	S
Andrena (*Micrandrena*) *piperi* Viereck, 1904	51	
Andrena (*Onagrandrena*) *anograe knowltoni* Linsley and MacSwain, 1961	30	
Andrena (*Onagrandrena*) *linsleyana* Thorp, 1987	26	CP
Andrena (*Parandrena*) *andrenoides* (Cresson, 1878)	84	
Andrena (*Parandrena*) *papagorum* Viereck & Cockerell, 1914	3	
Andrena (*Parandrena*) *wellesleyana* Robertson, 1897	32	
Andrena (*Plastandrena*) *prunorum* Cockerell, 1896	534	
Andrena (*Rhaphandrena*) *prima* Casad, 1896	9	
Andrena (*Scaphandrena*) *bruneri* Viereck & Cockerell, 1914	82	
Andrena (*Scaphandrena*) *chapmanae* Viereck, 1904	31	
Andrena (*Scaphandrena*) *cruciferarum* Ribble, 1974	1	NS
Andrena (*Scaphandrena*) *hicksi* Cockerell, 1925	2	
Andrena (*Scaphandrena*) *kaibabensis* Ribble, 1974	47	CP
Andrena (*Scaphandrena*) *merriami* Cockerell, 1901	13	
Andrena (*Scaphandrena*) *montrosensis* Viereck and Cockerell, 1914	12	
Andrena (*Scaphandrena*) *nigricula* LaBerge & Bouseman, 1977	80	
Andrena (*Scaphandrena*) *scurra* Viereck, 1904	40	
Andrena (*Scaphandrena*) *sieverti* Cockerell, 1906	6	
Andrena (*Scaphandrena*) n. sp. (aff. *kaibabensis*)	16	
Andrena (*Scrapteropsis*) *imitatrix* Cresson, 1872	1	
Andrena (*Simandrena*) *angustitarsata* Viereck, 1904	13	
Andrena (*Thysandrena*) *medionitens* Cockerell, 1902	413	
Andrena (*Thysandrena*) *w-scripta* Viereck, 1904	178	
Andrena (*Thysandrena*) sp. 7 (aff. *medionitens*)	15	
Andrena (*Thysandrena*) sp. 8 (aff. *w-scripta*)	10	
Andrena (*Trachandrena*) *cupreotincta* Cockerell, 1901	418	
Andrena (*Trachandrena*) *forbesii* Robertson, 1891	1	
Andrena (*Trachandrena*) *salicifloris* Cockerell, 1897	1	S
Andrena (*Trachandrena*) *striatifrons* Cockerell, 1897	32	
Andrena (*Trachandrena*) *zionensis* LaBerge, 1973	234	W
Andrena (*Tylandrena*) *hallii* Dunning, 1898	32	
Andrena (*Tylandrena*) *subaustralis* Cockerell, 1898	52	

Panurginae

Calliopsis, 4 species		
Calliopsis (*Calliopsima*) *chlorops* Cockerell, 1899	116	
Calliopsis (*Calliopsima*) *philiphunteri* Shinn and Engel, 2003	32	
Calliopsis (*Nomadopsis*) *puellae* (Cockerell, 1933)	35	
Calliopsis (*Nomadopsis*) *timberlakei* (Rozen, 1958)	19	
Macrotera, 4 species, 1 new species		
Macrotera (*Macroterella*) *opacella* (Timberlake, 1956)	61	
Macrotera (*Macroteropsis*) *arcuata* (Fox, 1893)	1	
Macrotera (*Macroteropsis*) *latior* (Cockerell, 1896)	1	
Macrotera (*Macroteropsis*) n. sp. (aff. *portalis*)	3	
Panurginus, 2 species		
Panurginus sp. 1	1	
Panurginus sp. 2	6	
Perdita, 87 species, 16 new species		
Perdita (*Allomacrotera*) *moabensis* Timberlake, 1971	36	CP
Perdita (*Cockerellia*) *albipennis* Cresson, 1868	81	
Perdita (*Cockerellia*) *coreopsidis kansensis* Timberlake, 1953	1	
Perdita (*Cockerellia*) *imbellis* Timberlake, 1968	74	CP
Perdita (*Cockerellia*) *lingualis* Cockerell, 1896	3	S
Perdita (*Cockerellia*) *perpulchra* Cockerell, 1896	121	
Perdita (*Cockerellia*) *verbesinae* Cockerell, 1896	12	
Perdita (*Epimacrotera*) *crassula* Timberlake, 1958	2	
Perdita (*Glossoperdita*) n. sp. 14 (aff. *giliae*)	164	
Perdita (*Hexaperdita*) *asteris* Cockerell, 1896	81	
Perdita (*Hexaperdita*) *heterothecae* Cockerell, 1900	18	NS
Perdita (*Perdita*) *aridella* Timberlake, 1960	1095	
Perdita (*Perdita*) *calloleuca* Cockerell, 1922	5859	
Perdita (*Perdita*) *croceipes* Timberlake, 1960	11	N
Perdita (*Perdita*) *crotonis juabensis* Timberlake, 1962	1	
Perdita (*Perdita*) *depressa* Timberlake, 1968	133	
Perdita (*Perdita*) *dubia parilis* Timberlake, 1958	120	
Perdita (*Perdita*) *electa* Timberlake, 1960	67	
Perdita (*Perdita*) *euzonata* Timberlake, 1964	6	
Perdita (*Perdita*) *fallax* Cockerell, 1896	300	
Perdita (*Perdita*) *festiva* Timberlake, 1958	898	CP
Perdita (*Perdita*) *glabrescens* Timberlake, 1962	1	NS
Perdita (*Perdita*) *hirsuta* Cockerell, 1896	3	NS, MD
Perdita (*Perdita*) *holoxantha* Timberlake, 1962	11	CP
Perdita (*Perdita*) *idonea* Timberlake, 1968	56	
Perdita (*Perdita*) *knowltoni* Timberlake, 1960	268	CP

Perdita (*Perdita*) *labergei* Timberlake, 1960	54	
Perdita (*Perdita*) *lepidosparti* Timberlake, 1958	7	
Perdita (*Perdita*) *luteola* Cockerell, 1894	383	
Perdita (*Perdita*) *mesillensis* Timberlake, 1968	23	NS
Perdita (*Perdita*) *morula* Timberlake, 1980	200	
Perdita (*Perdita*) *munda* Timberlake, 1958	61	CP
Perdita (*Perdita*) *nasuta obscurescens* Timberlake, 1962	55	
Perdita (*Perdita*) *nuda* Cockerell, 1896	199	
Perdita (*Perdita*) *phymatae* Cockerell, 1895	706	
Perdita (*Perdita*) *rectangulata* Cockerell, 1896	115	NS
Perdita (*Perdita*) *salicis imperialis* Cockerell, 1925	67	
Perdita (*Perdita*) *salicis salicis* Cockerell, 1896	97	
Perdita (*Perdita*) *salicis subtristis* Cockerell, 1933	128	
Perdita (*Perdita*) *similis* Timberlake, 1958	1533	
Perdita (*Perdita*) *subfasciata* Cockerell, 1897	2617	
Perdita (*Perdita*) *tortifoliae* Cockerell, 1906	350	
Perdita (*Perdita*) *vestita* Timberlake, 1958	263	CP
Perdita (*Perdita*) *wilmattae miriconis* Cockerell, 1922	18	GB
Perdita (*Perdita*) *xanthochroa* Timberlake, 1960	198	CP
Perdita (*Perdita*) *xerophila discrepans* Timberlake, 1962	64	E, MD
Perdita (*Perdita*) *zebrata flavens* Timberlake, 1958	2663	
Perdita (*Perdita*) *zebrata zebrata* Cresson, 1878	85	
Perdita (*Perdita*) sp. 2 (aff. *xanthochroa*)	7	
Perdita (*Perdita*) sp. 3 (aff. *varley*)	1	
Perdita (*Perdita*) sp. 4 (aff. *punctifera*)	5	
Perdita (*Perdita*) sp. 5 (aff. *munda*)	103	
Perdita (*Perdita*) sp. 6 (aff. *luteiventris*)	13	
Perdita (*Perdita*) sp. 7 (aff. *lateralis*)	33	
Perdita (*Perdita*) sp. 8 (aff. *koebelei*)	3	
Perdita (*Perdita*) sp. 9 (aff. *fuscipes*)	35	
Perdita (*Perdita*) sp. 10 (aff. *aridella*)	422	
Perdita (*Perdita*) sp. 11 (aff. *apacheorum*)	41	
Perdita (*Perdita*) sp. E1	171	
Perdita (*Perdita*) sp. E4	25	
Perdita (*Perdita*) sp. E8	1	
Perdita (*Perdita*) n. sp. 1	270	
Perdita (*Perdita*) n. sp. 2 (aff. *zebrata*)	166	
Perdita (*Perdita*) n. sp. 3 (aff. *tortifoliae*)	1	
Perdita (*Perdita*) n. sp. 4 (aff. *subfasciata*)	145	
Perdita (*Perdita*) n. sp. 5 (aff. *sphaeralceae*)	15	
Perdita (*Perdita*) n. sp. 6 (aff. *luteola*)	1	

Perdita (*Perdita*) n. sp. 7 (aff. *laticincta*)	6
Perdita (*Perdita*) n. sp. 8 (aff. *laticincta*)	2
Perdita (*Perdita*) n. sp. 9 (aff. *gerhardi*)	2
Perdita (*Perdita*) n. sp. 10 (aff. *eremica*)	192
Perdita (*Perdita*) n. sp. 11 (aff. *elongaticeps*)	1
Perdita (*Perdita*) n. sp. 12 (aff. *cuspidata*)	13
Perdita (*Perdita*) n. sp. 13 (aff. *confusa*)	157
Perdita (*Perdita*) n. sp. 14 (aff. *zonalis*)	12
Perdita (*Perdita*) n. sp. 15	103
Perdita (*Procockerellia*) *albonotata* Timberlake, 1954	26
Perdita (*Pygoperdita*) *duplonotata* Timberlake, 1956	527
Perdita (*Pygoperdita*) *fallugiae* Timberlake, 1956	11
Perdita (*Pygoperdita*) *mormonica* Timberlake, 1956	3
Perdita (*Pygoperdita*) sp. 1 (aff. *mohavensis*)	7
Perdita (*Xeromacrotera*) *cephalotes* (Cresson, 1878)	252
Perdita sp. E2	8
Perdita sp. E5	7
Perdita sp. E3	1
Perdita sp. E9	27
Perdita sp. E10	5
Perdita sp. E11	1
Perdita sp. E12	2
Perdita sp. E13	5
Pseudopanurgus, 12 species, 2 new species	
Pseudopanurgus (*Heterosarus*) *bakeri* (Cockerell, 1896)	1
Pseudopanurgus (*Heterosarus*) n. sp.	64
Pseudopanurgus (*Heterosarus*) sp. E1	6
Pseudopanurgus (*Heterosarus*) sp. E2	11
Pseudopanurgus (*Heterosarus*) sp. E3	62
Pseudopanurgus (*Pterosarus*) *irregularis* (Cockerell, 1922)	158
Pseudopanurgus (*Pterosarus*) sp. 1 (aff. *porterae*)	1
Pseudopanurgus (*Pterosarus*) sp. E1	36
Pseudopanurgus (*Pterosarus*) sp. E2	6
Pseudopanurgus (*Pterosarus*) sp. E3	36
Pseudopanurgus (*Pterosarus*) sp. E4	1
Pseudopanurgus (*Pterosarus*) n. sp. (aff. *irregularis*)	377
**Apidae, 158 species, 22 genera, 4 new species**	
Apinae	
Anthophora, 22 species, 3 new species	
Anthophora (*Anthophoroides*) n. sp. 1	7
Anthophora (*Anthophoroides*) n. sp. 2	114

Anthophora (*Anthophoroides*) *pueblo* Orr, 2016	2	CP
Anthophora (*Heliophila*) *albata* Cresson, 1876	271	
Anthophora (*Lophanthophora*) *affabilis* Cresson, 1878	39	
Anthophora (*Lophanthophora*) *dammersi* Timberlake, 1937	26	
Anthophora (*Lophanthophora*) *neglecta* Timberlake and Cockerell, 1936	130	
Anthophora (*Lophanthophora*) *pacifica* Cresson, 1878	41	
Anthophora (*Lophanthophora*) *porterae* Cockerell, 1900	19	
Anthophora (*Lophanthophora*) *ursina* Cresson, 1869	26	
Anthophora (*Melea*) *bomboides* Kirby, 1838	57	
Anthophora (*Melea*) *occidentalis* Cresson, 1869	59	
Anthophora (*Micranthophora*) *albata* Cresson, 1876	2	
Anthophora (*Micranthophora*) *curta* Provancher, 1895	7	
Anthophora (*Micranthophora*) *escalante* Orr, 2017	4	CP
Anthophora (*Micranthophora*) *peritomae* Cockerell, 1905	390	
Anthophora (*Micranthophora*) *petrophila* Cockerell, 1905	747	
Anthophora (*Mystacanthophora*) *montana* Cresson, 1869	26	
Anthophora (*Mystacanthophora*) *urbana* Cresson, 1878	1326	
Anthophora (*Pyganthophora*) *edwardsii* Cresson, 1878	1	
Anthophora (*Pyganthophora*) *lesquerellae* (Cockerell, 1896)	81	
Anthophora (*Pyganthophora*) n. sp.	17	
Anthophorula, 2 species		
Anthophorula (*Anthophorula*) *albata* (Timberlake, 1947)	30	
Anthophorula (*Anthophorula*) *crenulata* (Timberlake, 1980)	38	
Apis, 1 species		
Apis mellifera Linnaeus, 1758	2474	
Bombus, 6 species		
Bombus (*Bombias*) *nevadensis* Cresson, 1874	11	
Bombus (*Bombus*) *occidentalis* Greene, 1858	6	
Bombus (*Thoracobombus*) *fervidus* (Fabricius, 1798)	27	
Bombus (*Pyrobombus*) *huntii* Greene, 1860	183	
Bombus (*Pyrobombus*) *melanopygus* Nylander, 1848	49	
Bombus (*Cullumanobombus*) *morrisoni* Cresson, 1878	492	
Diadasia, 8 species		
Diadasia australis (Cresson, 1878)	166	
Diadasia diminuta (Cresson, 1878)	599	
Diadasia enavata (Cresson, 1872)	52	
Diadasia lutzi Cockerell, 1924	135	
Diadasia martialis Timberlake, 1940	10	NS
Diadasia ochracea (Cockerell, 1903)	16	
Diadasia rinconis Cockerell, 1897	11	

Diadasia vallicola Timberlake, 1940	1	N
Eucera, 11 species		
Eucera (*Synhalonia*) *acerba* (Cresson, 1879)	8	
Eucera (*Synhalonia*) *edwardsii* (Cresson, 1878)	4	
Eucera (*Synhalonia*) *frater* (Cresson, 1878)	128	S
Eucera (*Synhalonia*) *fulvitarsis* (Cresson, 1878)	160	
Eucera (*Synhalonia*) *lunata* (Timberlake, 1969)	6	N, MD
Eucera (*Synhalonia*) *mohavensis* (Timberlake, 1969)	44	
Eucera (*Synhalonia*) *phaceliae* (Cockerell, 1911)	71	
Eucera (*Synhalonia*) *primaveris* (Timberlake, 1969)	34	
Eucera (*Synhalonia*) *quadricincta* (Timberlake, 1969)	6	
Eucera (*Synhalonia*) *speciosa* (Cresson, 1878)	8	
Eucera (*Synhalonia*) *territella* (Cockerell, 1905)	87	
Exomalopsis, 1 species		
Exomalopsis (*Phanomalopsis*) *solidaginis* Cockerell, 1898	244	
Habropoda, 3 species		
Habropoda cineraria (Smith, 1879)	77	
Habropoda excellens (Timberlake, 1962)	73	
Habropoda morrisoni (Cresson, 1878)	236	
Melissodes, 27 species		
Melissodes (*Callimelissodes*) *coloradensis* Cresson, 1878	2	
Melissodes (*Callimelissodes*) *compositus* Tucker, 1909	1	
Melissodes (*Callimelissodes*) *glenwoodensis* Cockerell, 1905	172	
Melissodes (*Eumelissodes*) *agilis* Cresson, 1878	93	
Melissodes (*Eumelissodes*) *bicoloratus* LaBerge, 1961	38	
Melissodes (*Eumelissodes*) *bimatris* LaBerge, 1961	378	
Melissodes (*Eumelissodes*) *brevipyga* LaBerge, 1961	2	
Melissodes (*Eumelissodes*) *grindeliae* Cockerell, 1898	171	
Melissodes (*Eumelissodes*) *illatus* Lovell and Cockerell, 1906	1	
Melissodes (*Eumelissodes*) *lutulentus* LaBerge, 1961	79	
Melissodes (*Eumelissodes*) *menuachus* Cresson, 1868	17	
Melissodes (*Eumelissodes*) *montanus* Cresson, 1878	9	
Melissodes (*Eumelissodes*) *pallidisignatus* Cockerell, 1905	105	
Melissodes (*Eumelissodes*) *perlusus* Cockerell, 1925	48	
Melissodes (*Eumelissodes*) *perpolitus* LaBerge, 1961	63	
Melissodes (*Eumelissodes*) *rustica* (Kirby, 1802)	357	
Melissodes (*Eumelissodes*) *saponellus* Cockerell, 1908	28	
Melissodes (*Eumelissodes*) *semilupinus* Cockerell, 1905	133	
Melissodes (*Eumelissodes*) *snowii* Cresson, 1878	9	
Melissodes (*Eumelissodes*) *subagilis* Cockerell, 1905	4	
Melissodes (*Eumelissodes*) *submenuacha* Cockerell 1897	4	

Melissodes (*Eumelissodes*) *tristis* Cockerell, 1894	495	
Melissodes (*Eumelissodes*) *utahensis* LaBerge, 1961	122	
Melissodes (*Eumelissodes*) *verbesinarum* Cockerell, 1905	45	
Melissodes (*Tachymelissodes*) *dagosus* Cockerell, 1909	5	
Melissodes sp. E1	6	
Melissodes sp. M1	43	
Svastra, 2 species		
Svastra (*Epimelissodes*) *helianthelli* (Cockerell, 1905)	6	
Svastra (*Epimelissodes*) *obliqua expurgata* (Cockerell, 1925)	7	
Zacosmia, 1 species		
Zacosmia maculata (Cresson, 1879)	27	
Melecta, 5 species		
Melecta (*Melecta*) *alexanderi* Griswold and Parker, 1999	1	
Melecta (*Melecta*) *bohartorum* Linsley, 1939	2	
Melecta (*Melecta*) *pacifica* Cresson, 1878	3	
Melecta (*Melecta*) *separata separata* Cresson, 1879	4	
Melecta (*Melecta*) *thoracica* Cresson, 1875	73	
Xeromelecta, 1 species		
Xeromelecta (*Melectomorpha*) *californica* (Cresson, 1878)	29	
Nomadinae		
Epeolus, 5 species		
Epeolus mesillae mesillae (Cockerell, 1895)	6	
Epeolus minimus (Robertson, 1902)	56	
Epeolus pusillus Cresson, 1864	37	
Epeolus scutellaris Say, 1824	4	
Epeolus sp. 3	1	
Hexepeolus, 1 species		NG, MD
Hexepeolus rhodogyne Linsley and Michener, 1937	1	
Holcopasites, 1 species		
Holcopasites pulchellus (Cresson, 1878)	8	
Neolarra, 5 species		
Neolarra (*Neolarra*) *penicula* Shanks, 1978	6	
Neolarra (*Neolarra*) sp. 1	22	
Neolarra (*Neolarra*) *verbesinae* (Cockerell, 1895)	7	
Neolarra (*Phileremulus*) *cockerelli* (Crawford, 1916)	11	
Neolarra (*Phileremulus*) *vigilans* (Cockerell, 1895)	8	
Nomada, 34 species, 1 new species		
Nomada (*Centrias*) *crotchii* Cresson, 1878	18	
Nomada (*Centrias*) *munda* Cresson, 1878	2	
Nomada (*Centrias*) sp. E1	13	

Nomada (*Centrias*) sp. E2	8	
Nomada (*Holonomada*) *edwardsii* Cresson, 1878	39	
Nomada (*Holonomada*) *parkeri* Evans, 1972	9	
Nomada (*Laminomada*) n. sp.	3	
Nomada (*Micronomada*) sp. E1	4	
Nomada (*Micronomada*) sp. E2	4	
Nomada (*Micronomada*) sp. E3	1	
Nomada (*Micronomada*) sp. E4	5	
Nomada (*Nomada*) sp. *E*1	161	
Nomada (*Nomada*) sp. E2	17	
Nomada (*Nomada*) sp. E3	118	
Nomada (*Nomada*) sp. E4	153	
Nomada (*Nomada*) sp. E5	13	
Nomada (*Nomada*) sp. E6	4	
Nomada (*Nomada*) sp. E7	23	
Nomada (*Nomada*) sp. E8	40	
Nomada (*Nomada*) sp. E10	2	
Nomada (*Nomada*) sp. E11	28	
Nomada (*Nomada*) sp. E12	8	
Nomada (*Nomada*) sp. E13	1	
Nomada (*Nomada*) sp. E14	7	
Nomada (*Nomada*) sp. E15	18	
Nomada (*Nomada*) sp. E16	14	
Nomada (*Nomada*) sp. E17	4	
Nomada (*Nomada*) sp. E18	26	
Nomada (*Nomada*) sp. E19	3	
Nomada (*Nomada*) sp. E20	1	
Nomada (*Nomada*) sp. E22	11	
Nomada (*Nomada*) sp. E23	4	
Nomada (*Nomada*) sp. E25	1	
Nomada (*Nomadita*) *mutans* Cockerell, 1910	5	
Oreopasites, 1 species		
Oreopasites sp.	1	
Paranomada, 1 species		
Paranomada sp. 1 (aff. *nitida*)	1	NG, SD
Triepeolus, 14 species		
Triepeolus balteatus Cockerell, 1921	26	S
Triepeolus dacotensis (Stevens, 1919)	3	
Triepeolus denverensis Cockerell, 1910	4	
Triepeolus diversipes Cockerell, 1924	3	
Triepeolus eldoradensis (Cockerell, 1910)	16	

Triepeolus helianthi (Robertson, 1897)	4	
Triepeolus micropygius Robertson, 1903	2	
Triepeolus norae Cockerell, 1907	1	
Triepeolus subalpinus Cockerell, 1910	21	
Triepeolus timberlakei Cockerell, 1929	34	
Triepeolus sp. 42	6	
Triepeolus sp. 51	1	
Triepeolus sp. 69	5	
Triepeolus sp. 76	1	
Xylocopinae		
Ceratina, 4 species		
Ceratina (*Zadontomerus*) *apacheorum* Daly, 1973	265	
Ceratina (*Zadontomerus*) *nanula* Cockerell, 1897	5188	
Ceratina (*Zadontomerus*) *neomexicana* Cockerell, 1901	608	
Ceratina (*Zadontomerus*) *pacifica* H. S. Smith, 1907	678	
Xylocopa, 2 species		
Xylocopa (*Xylocopoides*) *californica* Cresson, 1864	199	
Xylocopa (*Notoxylocopa*) *tabaniformis androleuca* Michener, 1940	27	
**Colletidae, 34 species, 2 genera, 6 new species**		
Colletinae		
Colletes, 22 species, 3 new species		
Colletes compactus Cresson, 1868	43	
Colletes daleae Cockerell, 1897	687	
Colletes eulophi Robertson, 1891	70	
Colletes fulgidus fulgidus Swenk, 1904	1	
Colletes gypsicolens Cockerell, 1897	416	
Colletes intermixtus Swenk, 1905	2	
Colletes kincaidii Cockerell, 1898	1	
Colletes larreae Timberlake, 1951	73	
Colletes laticinctus Timberlake, 1951	33	
Colletes louisae Cockerell, 1897	265	
Colletes lutzi lutzi Timberlake, 1943	6	
Colletes mandibularis Smith, 1853	9	
Colletes petalostemonis Swenk, 1906	178	W
Colletes phaceliae Cockerell, 1906	1359	
Colletes simulans nevadensis Swenk, 1908	96	
Colletes slevini Cockerell, 1925	503	
Colletes sphaeralceae Timberlake, 1951	316	
Colletes sp. 1	57	
Colletes sp. 2 (aff. *petalostemonis*)	32	

Colletes n. sp. 1 (aff. *algarobiae*)	2
Colletes n. sp. 2 (aff. *aberrans*)	6
Colletes n. sp. 3	4
Hylaeinae	
Hylaeus, 12 species, 3 new species	
Hylaeus (*Hylaeus*) *leptocephalus* (Morawitz, 1871)	16
Hylaeus (*Hylaeus*) *granulatus* (Metz, 1911)	6
Hylaeus (*Hylaeus*) *mesillae cressoni* (Cockerell, 1907)	605
Hylaeus (*Hylaeus*) *rudbeckiae* (Cockerell and Casad, 1895)	2
Hylaeus (*Hylaeus*) *verticalis* (Cresson, 1869)	1
Hylaeus (*Hylaeus*) sp. 1 (aff. *mesillae*)	195
Hylaeus (*Paraprosopis*) *coloradensis* (Cockerell, 1896)	2
Hylaeus (*Paraprosopis*) *megalotis* (Swenk and Cockerell, 1910)	929
Hylaeus (*Paraprosopis*) *wootoni* (Cockerell, 1896)	494
Hylaeus (*Paraprosopis*) n. sp. 1 (aff. *cookii*)	1228
Hylaeus (*Paraprosopis*) n. sp. 2	129
Hylaeus (*Prosopis*) n. sp. 3	1010
**Halictidae, 114 species, 7 genera, 2 new species**	
Halictinae	
Agapostemon, 5 species, 1 new species	
Agapostemon (*Agapostemon*) *angelicus* Cockerell, 1924	231*
Agapostemon (*Agapostemon*) *texanus* Cresson, 1872	185*
Agapostemon (*Agapostemon*) *femoratus* Crawford, 1901	2
Agapostemon (*Agapostemon*) *melliventris* Cresson, 1874	15
Agapostemon (*Agapostemon*) n. sp. (aff. *splendens*)	7
Halictus, 5 species	
Halictus (*Nealictus*) *farinosus* Smith, 1853	170
Halictus (*Odontalictus*) *ligatus* Say, 1837	33
Halictus (*Protohalictus*) *rubicundus* (Christ, 1791)	12
Halictus (*Seladonia*) *confusus* Smith, 1853	8
Halictus (*Seladonia*) *tripartitus* Cockerell, 1895	3676
Lasioglossum, 76 species	
Lasioglossum (*Dialictus*) *abundipunctum* Gibbs, 2010	208
Lasioglossum (*Dialictus*) *albohirtum* (Crawford, 1907)	581
Lasioglossum (*Dialictus*) *aliud* (Sandhouse, 1924)	28
Lasioglossum (*Dialictus*) *brunneiventre* (Crawford, 1907)	7
Lasioglossum (*Dialictus*) *clarissimum* (Ellis, 1914)	9
Lasioglossum (*Dialictus*) *clematisellum* (Cockerell, 1904)	557
Lasioglossum (*Dialictus*) *griswoldi* Gibbs, 2009	1
Lasioglossum (*Dialictus*) *hudsoniellum* (Cockerell, 1919)	86

Lasioglossum (*Dialictus*) *hyalinum* (Crawford, 1907)	124
Lasioglossum (*Dialictus*) *impavidum* (Sandhouse, 1924)	161
Lasioglossum (*Dialictus*) *incompletum* (Crawford, 1907)	1
Lasioglossum (*Dialictus*) *microlepoides* (Ellis, 1914)	8
Lasioglossum (*Dialictus*) *nevadense* (Crawford, 1907)	892
Lasioglossum (*Dialictus*) *pallidellum* (Ellis, 1914)	22
Lasioglossum (*Dialictus*) *perdifficile* (Cockerell, 1895)	111
Lasioglossum (*Dialictus*) *perparvum* (Ellis, 1914)	5
Lasioglossum (*Dialictus*) *petrellum* (Cockerell, 1903)	45
Lasioglossum (*Dialictus*) *prasinogaster* Gibbs, 2010	9
Lasioglossum (*Dialictus*) *pruinosum* (Robertson, 1892)	143
Lasioglossum (*Dialictus*) *ruidosense* (Cockerell, 1897)	275
Lasioglossum (*Dialictus*) *sedi* (Sandhouse, 1924)	1
Lasioglossum (*Dialictus*) *semibrunneum* (Cockerell, 1895)	10
Lasioglossum (*Dialictus*) *semicaeruleum* (Cockerell, 1895)	146
Lasioglossum (*Dialictus*) *tegulare* (Robertson, 1890)	88
Lasioglossum (*Dialictus*) *tegulariforme* (Crawford, 1907)	41
Lasioglossum (*Dialictus*) sp. 1 (aff. *albuquerquense*)	164
Lasioglossum (*Dialictus*) sp. 2 (aff. *aliud*)	29
Lasioglossum (*Dialictus*) sp. 3 (aff. *hudsoniellum*)	14
Lasioglossum (*Dialictus*) sp. 4 (aff. *hyalinum*)	21
Lasioglossum (*Dialictus*) sp. 5 (aff. *impavidum*)	86
Lasioglossum (*Dialictus*) sp. 6 (aff. *pacatum*)	3
Lasioglossum (*Dialictus*) sp. 7 (aff. *hunteri*)	13
Lasioglossum (*Dialictus*) sp. 8 (aff. *nevadense*)	27
Lasioglossum (*Dialictus*) sp. 9 (aff. *pruinosum*)	2
Lasioglossum (*Dialictus*) sp. A	57
Lasioglossum (*Dialictus*) sp. E1	57
Lasioglossum (*Dialictus*) sp. E2	34
Lasioglossum (*Dialictus*) sp. E3	3
Lasioglossum (*Dialictus*) sp. E5	7
Lasioglossum (*Dialictus*) sp. E6	52
Lasioglossum (*Dialictus*) sp. E7	11
Lasioglossum (*Dialictus*) sp. E8	7
Lasioglossum (*Dialictus*) sp. E9	59
Lasioglossum (*Dialictus*) sp. E10	4
Lasioglossum (*Dialictus*) sp. E11	38
Lasioglossum (*Dialictus*) sp. E12	1
Lasioglossum (*Dialictus*) sp. E14	1
Lasioglossum (*Dialictus*) sp. E16	5
Lasioglossum (*Dialictus*) sp. E18	4

Lasioglossum (*Dialictus*) sp. E19	14
Lasioglossum (*Dialictus*) sp. E22	158
Lasioglossum (*Dialictus*) sp. E23	51
Lasioglossum (*Dialictus*) sp. E24	52
Lasioglossum (*Dialictus*) sp. M2	5
Lasioglossum (*Dialictus*) sp. M3	5
Lasioglossum (*Dialictus*) sp. M11	4
Lasioglossum (*Dialictus*) sp. M15	16
Lasioglossum (*Dialictus*) sp. M17	52
Lasioglossum (*Evylaeus*) sp. E1	52
Lasioglossum (*Evylaeus*) sp. E2	180
Lasioglossum (*Evylaeus*) sp. E3 (aff. *nigrescens*)	1
Lasioglossum (*Evylaeus*) sp. E5	10
Lasioglossum (*Evylaeus*) sp. E7	14
Lasioglossum (*Hemihalictus s.l.*) *inconditum* (Cockerell, 1916)	285
Lasioglossum (*Hemihalictus s.l.*) *ovaliceps* (Cockerell, 1898)	112
Lasioglossum (*Hemihalictus s.l.*) *pectoraloides* (Cockerell, 1895)	4
Lasioglossum (*Hemihalictus s.l.*) *pulveris* (Cockerell, 1930)	395
Lasioglossum (*Hemihalictus s.l.*) *swenki* (Crawford, 1906)	21
Lasioglossum (*Lasioglossum*) *egregium* (Vachal, 1904)	179
Lasioglossum (*Lasioglossum*) *heterorhinum* (Cockerell, 1930)	12
Lasioglossum (*Lasioglossum*) *lampronotum* (Cameron, 1905)	70
Lasioglossum (*Lasioglossum*) *sisymbrii* (Cockerell, 1895)	368
Lasioglossum (*Sphecodogastra s.l.*) *cooleyi* (Crawford, 1906)	2
Lasioglossum (*Sphecodogastra*) *lusorium* (Cresson, 1872)	49
Lasioglossum (*Sphecodogastra s.l.*) *nigrescens* (Crawford, 1907)	14
Lasioglossum (*Sphecodogastra*) *noctivaga* Linsley and MacSwain, 1962	11
Sphecodes, 18 species	
Sphecodes sp. E1	11
Sphecodes sp. E2	323
Sphecodes sp. E3	54
Sphecodes sp. E4	2
Sphecodes sp. E5	5
Sphecodes sp. E6	5
Sphecodes sp. E7	52
Sphecodes sp. E8	178
Sphecodes sp. E9	2
Sphecodes sp. E10	1
Sphecodes sp. E11	1

Sphecodes sp. E12	6	
Sphecodes sp. E13	4	
Sphecodes sp. E14	29	
Sphecodes sp. E15	5	
Sphecodes sp. E16	2	
Sphecodes sp. E17	3	
Sphecodes sp. E18	16	
Nomiinae		
Dieunomia, 2 species		
Dieunomia (*Dieunomia*) *heteropoda* (Say, 1824)	11	
Dieunomia (*Epinomia*) *nevadensis* (Cresson, 1874)	300	
Nomia, 1 species		
Nomia (*Acunomia*) *tetrazonata tetrazonata* Cockerell, 1910	2	N
Rophitinae		
Dufourea, 7 species, 1 new species		
Dufourea contarovici Bohart, 1980	3	
Dufourea harveyi (Cockerell, 1906)	1	
Dufourea malacothricis Timberlake, 1939	138	
Dufourea sandhouseae (Michener, 1937)	34	
Dufourea tinsleyi (Cockerell, 1898)	5	
Dufourea n. sp. 1	8	
Dufourea sp. (aff. *saundersi*)	2	
**Megachilidae, 173 species, 15 genera, 12 new species**		
Megachilinae		
Anthidiellum, 1 species		
Anthidiellum (*Loyalanthidium*) *notatum* (Latreille, 1809)	89	
Anthidium, 12 species		
Anthidium (*Anthidium*) *atripes* Cresson, 1879	95	
Anthidium (*Anthidium*) *atripoides* Gonzalez and Griswold, 2013	55	
Anthidium (*Anthidium*) *cockerelli* Schwarz, 1928	34	
Anthidium (*Anthidium*) *dammersi* Cockerell, 1937	27	
Anthidium (*Anthidium*) *duomarginatum* Gonzalez and Griswold, 2013	1	
Anthidium (*Anthidium*) *emarginatum* (Say, 1824)	1	
Anthidium (*Anthidium*) *maculosum* Cresson, 1878	21	
Anthidium (*Anthidium*) *mormonum* Cresson, 1878	149	
Anthidium (*Anthidium*) *palmarum* Cockerell, 1904	6	
Anthidium (*Anthidium*) *placitum* Cresson, 1879	111	
Anthidium (*Anthidium*) *porterae* Cockerell, 1900	2	
Anthidium (*Anthidium*) *schwarzi* Gonzalez and Griswold,	2	

2013		
Ashmeadiella, 22 species, 3 new species		
Ashmeadiella (*Arogochila*) *australis* (Cockerell, 1902)	107	
Ashmeadiella (*Arogochila*) *cazieri* Michener, 1939	12	
Ashmeadiella (*Arogochila*) *erema* Michener, 1939	12	
Ashmeadiella (*Arogochila*) *lutzi* (Cockerell, 1930)	10	
Ashmeadiella (*Arogochila*) n. sp. 1 (aff. *leachi*)	2	
Ashmeadiella (*Arogochila*) n. sp. 2 (aff. *micheneri*)	76	
Ashmeadiella (*Arogochila*) n. sp. 3 (aff. *salviae*)	1	
Ashmeadiella (*Ashmeadiella*) *aridula* Cockerell, 1910	703	
Ashmeadiella (*Ashmeadiella*) *bucconis* (Say, 1837)	547	
Ashmeadiella (*Ashmeadiella*) *cactorum* (Cockerell, 1897)	678	
Ashmeadiella (*Ashmeadiella*) *californica* (Ashmead, 1897)	17	
Ashmeadiella (*Ashmeadiella*) *cubiceps clypeata* Michener, 1936	10	NS
Ashmeadiella (*Ashmeadiella*) *difugita* Michener, 1939	1	
Ashmeadiella (*Ashmeadiella*) *foveata* Michener, 1939	19	
Ashmeadiella (*Ashmeadiella*) *gillettei* Titus, 1904	244	
Ashmeadiella (*Ashmeadiella*) *meliloti* (Cockerell, 1897)	192	
Ashmeadiella (*Ashmeadiella*) *opuntiae* (Cockerell, 1897)	464	
Ashmeadiella (*Ashmeadiella*) *sonora* Michener, 1939	103	
Ashmeadiella (*Ashmeadiella*) *vandykiella* Michener, 1949	40	NS
Ashmeadiella (*Ashmeadiella*) sp. 1 (aff. *rufipes*)	4	
Ashmeadiella (*Ashmeadiella*) sp. 2 (aff. *titusi*)	11	
Ashmeadiella (*Cubitognatha*) *xenomastax* Michener, 1939	48	
Atoposmia, 4 species, 3 new species		
Atoposmia (*Atoposmia*) sp. 1 (aff. *triodonta*)	4	
Atoposmia (*Atoposmia*) n. sp. 1	48	
Atoposmia (*Atoposmia*) n. sp. 2 (aff. *anthodyta*)	11	
Atoposmia (*Eremosmia*) n. sp. 3 (aff. *daleae*)	47	
Coelioxys, 9 species		
Coelioxys (*Boreocoelioxys*) *rufitarsis* Smith, 1854	3	
Coelioxys (*Boreocoelioxys*) *sayi* Robertson, 1897	1	
Coelioxys (*Coelioxys*) *hirsutissimus* Cockerell, 1912	2	
Coelioxys (*Coelioxys*) *mitchelli* J. R. Baker, 1975	1	NS
Coelioxys (*Cyrtocoelioxys*) *gilensis* Cockerell, 1898	5	
Coelioxys (*Synocoelioxys*) *apacheorum* Cockerell, 1900	1	
Coelioxys (*Synocoelioxys*) *hunteri* Crawford, 1914	11	NS
Coelioxys (*Xerocoelioxys*) *grindeliae* Cockerell, 1900	13	
Coelioxys (*Xerocoelioxys*) *mesae* Cockerell, 1921	2	
Diathidium, 9 species		

Dianthidium (*Adanthidium*) *arizonicum* Rohwer, 1916	17	NS
Dianthidium (*Dianthidium*) *cressonii* (Dalla Torre, 1896)	55	
Dianthidium (*Dianthidium*) *dubium dubium* Schwarz, 1928	1	
Dianthidium (*Dianthidium*) *heterulkei heterulkei* Schwarz, 1940	1	
Dianthidium (*Dianthidium*) *implicatum* Timberlake, 1948	16	
Dianthidium (*Dianthidium*) *parvum* (Cresson, 1878)	349	
Dianthidium (*Dianthidium*) *platyurum platyurum* Cockerell, 1923	35	
Dianthidium (*Dianthidium*) *pudicum pudicum* (Cresson, 1879)	117	
Dianthidium (*Dianthidium*) *ulkei* (Cresson, 1878)	302	
Dioxys, 1 species		
Dioxys pomonae pomonae Cockerell, 1910	20	
Dioxys pomonae timberlakei Hurd, 1958	5	
Heriades, 4 species		
Heriades (*Neotrypetes*) *cressoni* Michener, 1938	357	
Heriades (*Neotrypetes*) *micropthalma* Michener, 1954	730	
Heriades (*Neotrypetes*) *timberlakei* Michener, 1938	732	
Heriades (*Neotrypetes*) *variolosa* (Cresson, 1872)	1	
Hoplitis, 9 species, 2 new species		
Hoplitis (*Alcidamea*) n. sp. 2	15	
Hoplitis (*Alcidamea*) *grinnelli* (Cockerell, 1910)	28	
Hoplitis (*Alcidamea*) *producta panamintana* Michener, 1947	15	
Hoplitis (*Cyrtosmia*) *hypocrita* (Cockerell, 1906)	12	
Hoplitis (*Dasyosmia*) *paroselae* Michener, 1947	13	
Hoplitis (*Monumetha*) *albifrons argentifrons* (Cresson, 1864)	28	
Hoplitis (*Penteriades*) *incanescens* (Cockerell, 1922)	82	CP, GSENM
Hoplitis (*Proteriades*) n. sp. 1 (aff. *shoshone*)	26	
Hoplitis (*Proteriades*) *zuni* (Parker, 1977)	229	
Lithurgopsis, 1 species		
Lithurgopsis apicalis (Cresson, 1875)	372	
Megachile, 33 species, 1 new species		
Megachile (*Argyropile*) *parallela* Smith, 1853	88	
Megachile (*Argyropile*) *rossi* Mitchell, 1943	9	
Megachile (*Argyropile*) *townsendiana* Cockerell, 1898	3	
Megachile (*Chelostomoides*) *prosopidis* Cockerell, 1900	136	
Megachile (*Chelostomoides*) *subexilis* Cockerell, 1908	78	
Megachile (*Eutricharea*) *rotundata* (Fabricius, 1787)	1	
Megachile (*Litomegachile*) *coquilletti* Cockerell, 1915	3	
Megachile (*Litomegachile*) *lippiae* Cockerell, 1900	84	

Megachile (*Litomegachile*) *mendica* Cresson, 1878	15	
Megachile (*Litomegachile*) *onobrychidis* Cockerell, 1908	5	
Megachile (*Litomegachile*) *texana* Cresson, 1878	132	
Megachile (*Litomegachile*) sp. 2 (aff. *brevis*)	93	
Megachile (*Megachile*) *montivaga* Cresson, 1878	10	
Megachile (*Megachiloides*) *anograe* Cockerell, 1908	14	S
Megachile (*Megachiloides*) *casadae* Cockerell, 1898	30	
Megachile (*Megachiloides*) *legalis* Cresson, 1879	61	
Megachile (*Megachiloides*) *manifesta* Cresson, 1878	12	CP
Megachile (*Megachiloides*) *micheneri* Mitchell, 1936	1	
Megachile (*Megachiloides*) *mucorosa* Cockerell, 1908	2	CP
Megachile (*Megachiloides*) *nevadensis* Cresson, 1879	341	
Megachile (*Megachiloides*) *subanograe* Mitchell, 1934	59	
Megachile (*Megachiloides*) *sublaurita* Mitchell, 1927	79	
Megachile (*Megachiloides*) *subnigra* Cresson, 1879	35	
Megachile (*Megachiloides*) sp. 1	1	
Megachile (*Megachiloides*) n. sp. (aff. *umatillensis*)	17	
Megachile (*Sayapis*) *fidelis* Cresson, 1878	9	
Megachile (*Sayapis*) *inimica sayi* (Cresson, 1878)	150	
Megachile (*Sayapis*) *mellitarsis* Cresson, 1878	1	
Megachile (*Sayapis*) *pugnata* Say, 1837	1	
Megachile (*Xanthosarus*) *agustini* Cockerell, 1905	28	CP
Megachile (*Xanthosarus*) *cochisiana* Mitchell, 1934	117	
Megachile (*Xanthosarus*) *perihirta* Cockerell, 1898	6	
Megachile (*Xeromegachile*) sp. 2	1	
Osmia, 44 species, 2 new species		
Osmia (*Acanthosmioides*) *alpestris* Rust and Bohart, 1986	4	
Osmia (*Acanthosmioides*) *austromaritima* Michener, 1936	7	S
Osmia (*Acanthosmioides*) *integra* Cresson, 1878	199	
Osmia (*Acanthosmioides*) *longula* Cresson, 1864	2	
Osmia (*Acanthosmioides*) *nigrifrons* Cresson, 1878	59	
Osmia (*Acanthosmioides*) *unca* Michener, 1937	30	
Osmia (*Cephalosmia*) *californica* Cresson, 1864	115	
Osmia (*Cephalosmia*) *grinnelli* Cockerell, 1910	38	
Osmia (*Cephalosmia*) *montana montana* Cresson, 1864	4	
Osmia (*Cephalosmia*) *subaustralis* Cockerell, 1900	1	
Osmia (*Helicosmia*) *coloradensis* Cresson, 1878	167	
Osmia (*Helicosmia*) *texana* Cresson, 1872	6	
Osmia (*Melanosmia*) *albolateralis* Cockerell, 1906	23	
Osmia (*Melanosmia*) *austromaritima* Michener, 1936	3	
Osmia (*Melanosmia*) *brevis* Cresson, 1864	199	

Osmia (*Melanosmia*) *bruneri* Cockerell, 1897	193	
Osmia (*Melanosmia*) *cerasi* Cockerell, 1897	16	
Osmia (*Melanosmia*) *clarescens* Cockerell, 1911	4	
Osmia (*Melanosmia*) *cobaltina* Cresson, 1878	152	
Osmia (*Melanosmia*) *crassa* Rust and Bohart, 1986	20	
Osmia (*Melanosmia*) *dakotensis* Michener, 1937	35	
Osmia (*Melanosmia*) *ednae* Cockerell, 1907	16	S
Osmia (*Melanosmia*) *gaudiosa* Cockerell, 1907	96	
Osmia (*Melanosmia*) *iridis* Cockerell and Titus, 1902	21	S
Osmia (*Melanosmia*) *kincaidii* Cockerell, 1897	175	S
Osmia (*Melanosmia*) *liogastra* Cockerell, 1933	6	
Osmia (*Melanosmia*) *marginata* Michener, 1936	6	
Osmia (*Melanosmia*) *phenax* Cockerell, 1897	45	
Osmia (*Melanosmia*) *prunorum* Cockerell, 1897	3	
Osmia (*Melanosmia*) *pusilla* Cresson, 1864	2	
Osmia (*Melanosmia*) *rawlinsi* Sandhouse, 1939	110	
Osmia (*Melanosmia*) *sanrafaelae* Parker, 1985	161	
Osmia (*Melanosmia*) *trevoris* Cockerell, 1897	193	
Osmia (*Melanosmia*) sp. 1 (aff. *cyanella*)	1	
Osmia (*Melanosmia*) sp. E1	1	
Osmia (*Melanosmia*) sp. E2	1	
Osmia (*Melanosmia*) sp. E3	1	
Osmia (*Melanosmia*) sp. E4	1	
Osmia (*Melanosmia*) sp. E5	3	
Osmia (*Melanosmia*) n. sp. 1	34	CP, GSENM
Osmia (*Melanosmia*) n. sp. 2 (aff. *enixa*)	19	
Osmia (*Osmia*) *lignaria propinqua* Cresson, 1864	342	
Osmia (*Osmia*) *ribifloris ribifloris* Cockerell, 1900	172	
Osmia (*Trichinosmia*) *latisulcata* Michener, 1936	17	
Protosmia, 1 species		
Protosmia (*Chelostomopsis*) *rubifloris* (Cockerell, 1898)	110	N, MD
Stelis, 16 species, 2 new species		
Stelis (*Dolichostelis*) *rudbeckiarum* Cockerell, 1904	12	
Stelis (*Stelis*) *anasazi* Parker and Griswold, 2013	5	
Stelis (*Stelis*) *carnifex* Cockerell, 1911	46	
Stelis (*Stelis*) *imperialis* Parker and Griswold, 2013	1	
Stelis (*Stelis*) *interrupta* Cresson, 1897	2	
Stelis (*Stelis*) *lamelliterga* Parker and Griswold, 2013	9	
Stelis (*Stelis*) *lateralis* Cresson, 1864	4	

Stelis (*Stelis*) *montana* Cresson, 1864	3
Stelis (*Stelis*) n. sp. 1	2
Stelis (*Stelis*) n. sp. 2	1
Stelis (*Stelis*) *occidentalis* Parker and Griswold, 2013	3
Stelis (*Stelis*) *paiute* Parker and Griswold, 2013	1
Stelis (*Stelis*) *palmarum* Timberlake, 1941	12
Stelis (*Stelis*) *pavonina* (Cockerell, 1908)	1
Stelis (*Stelis*) *robertsoni* Timberlake, 1941	2
Stelis (*Stelis*) *subemarginata* Cresson, 1878	1
Trachusa, 2 species	
Trachusa (*Heteranthidium*) *cordaticeps* (Michener, 1949)	4
Trachusa (*Heteranthidium*) *zebrata* (Cresson, 1872)	1
**Melittidae, 5 species, 1 genus, 2 new species**	
Hesperapis, 5 species, 2 new species	
Hesperapis (*Carinapis*) *carinata* Stevens, 1919	1
Hesperapis (*Carinapis*) *oliviae* (Cockerell, 1897)	26
Hesperapis (*Disparapis*) sp. 1 (aff. *cockerelli*)	33
Hesperapis (*Disparapis*) n. sp. 1 (aff. *disparapis*)	5
Hesperapis (*Panurgomia*) n. sp. 2	211
